# Supplementary material for: Association of vitamin D receptor mRNA expression, vitamin D deficiency and genetic variant in patients with multi-drug resistant pulmonary tuberculosis
Source: BMC Infect Dis. 2025 Oct 15;25:1334. doi: 10.1186/s12879-025-11707-7 (PMC12522335; doi:10.1186/s12879-025-11707-7)
Supplement: Supplementary file 1 — Supplementary Material 1. [file 12879_2025_11707_MOESM1_ESM.ppt]

## Slide 1
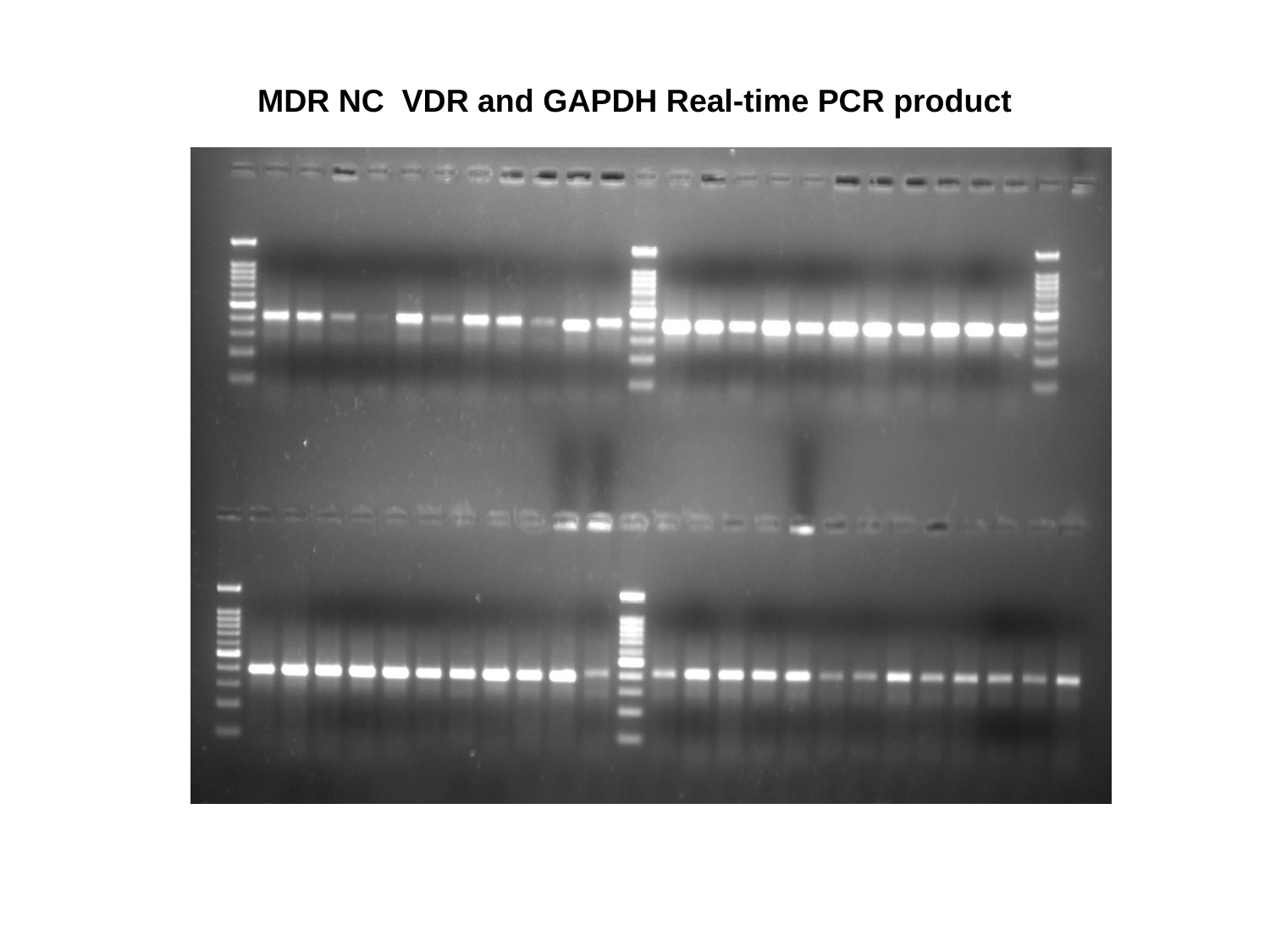

MDR NC VDR and GAPDH Real-time PCR product

## Slide 2
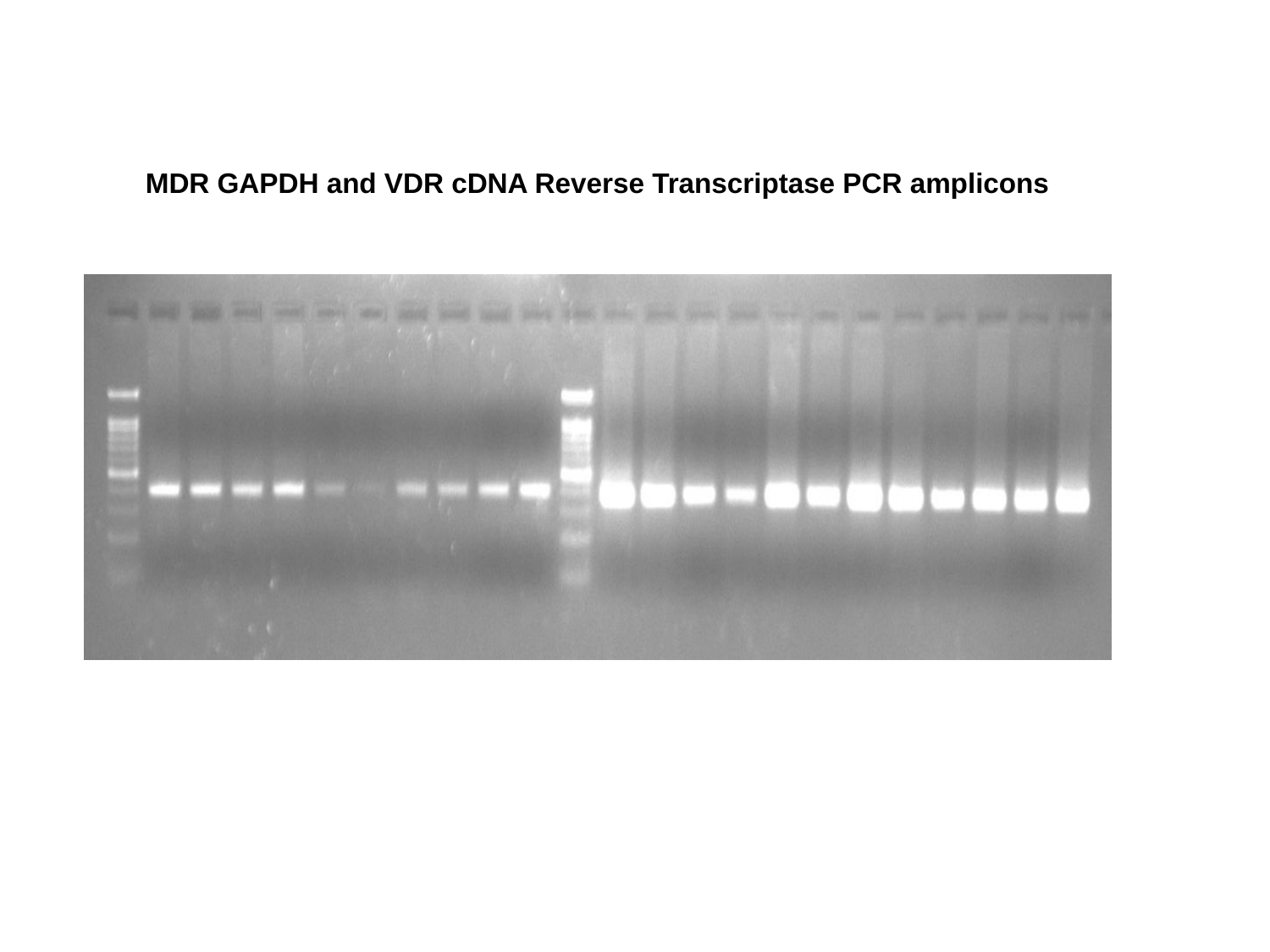

MDR GAPDH and VDR cDNA Reverse Transcriptase PCR amplicons

## Slide 3
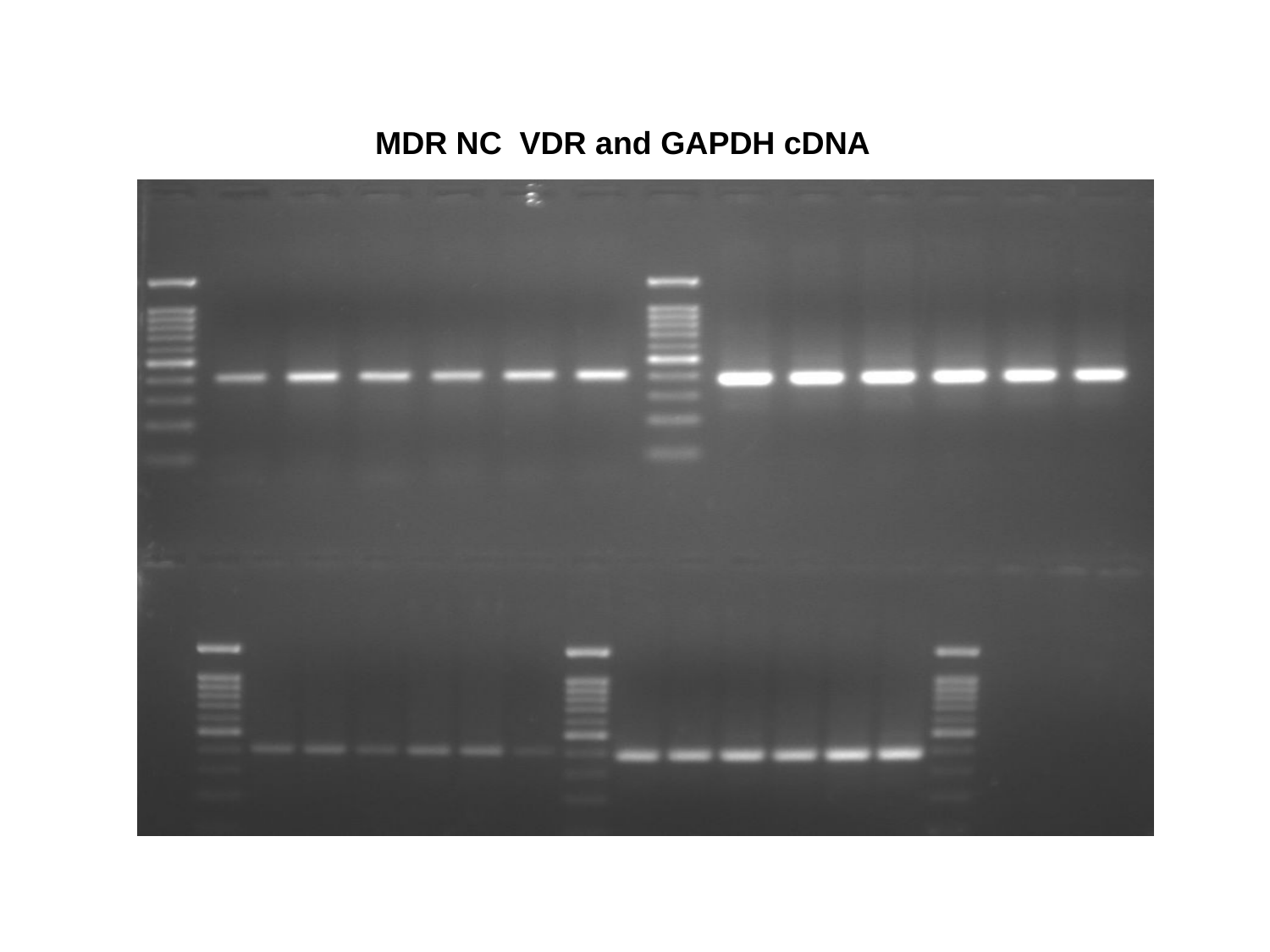

MDR NC VDR and GAPDH cDNA

## Slide 4
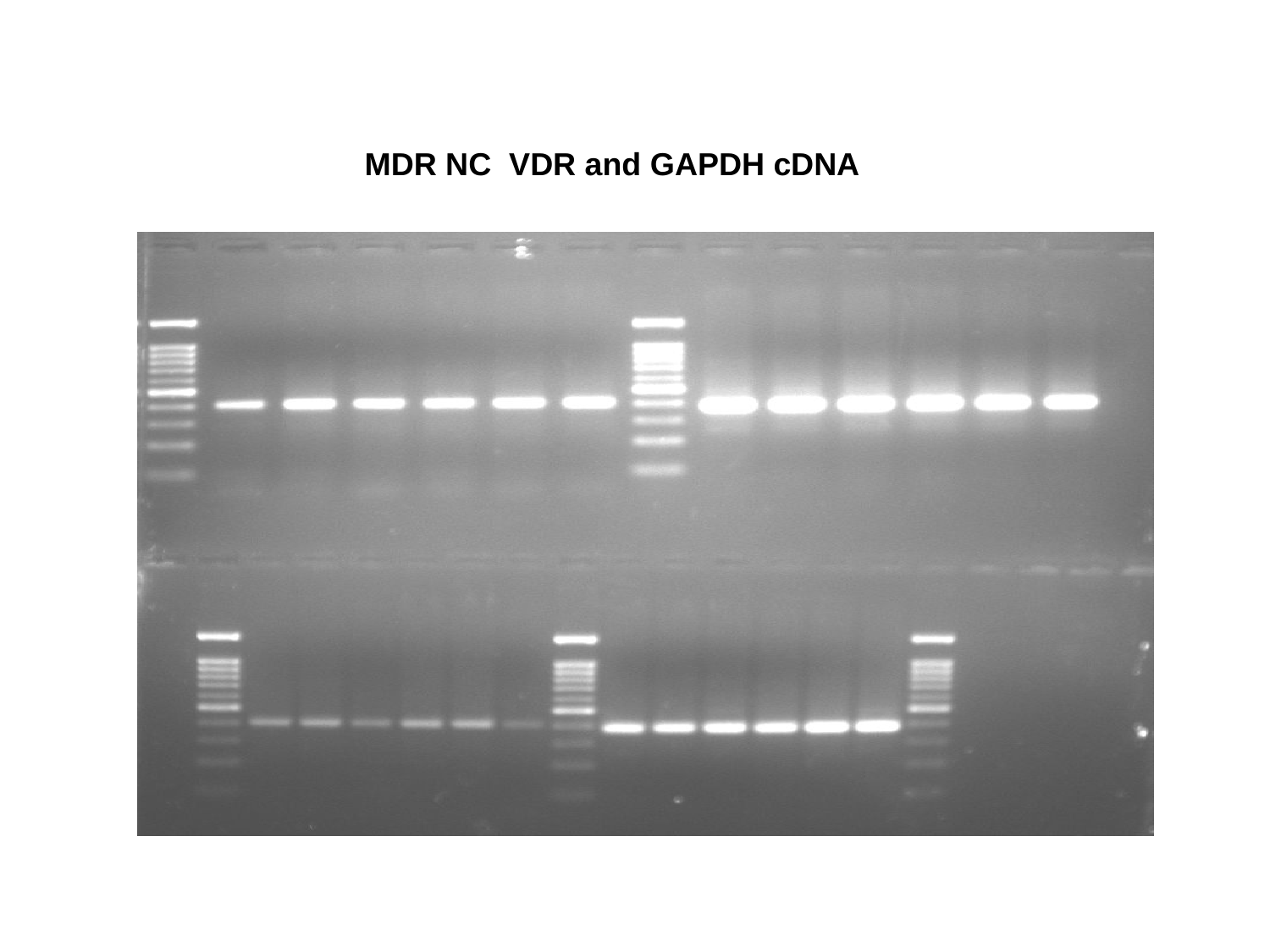

MDR NC VDR and GAPDH cDNA

## Slide 5
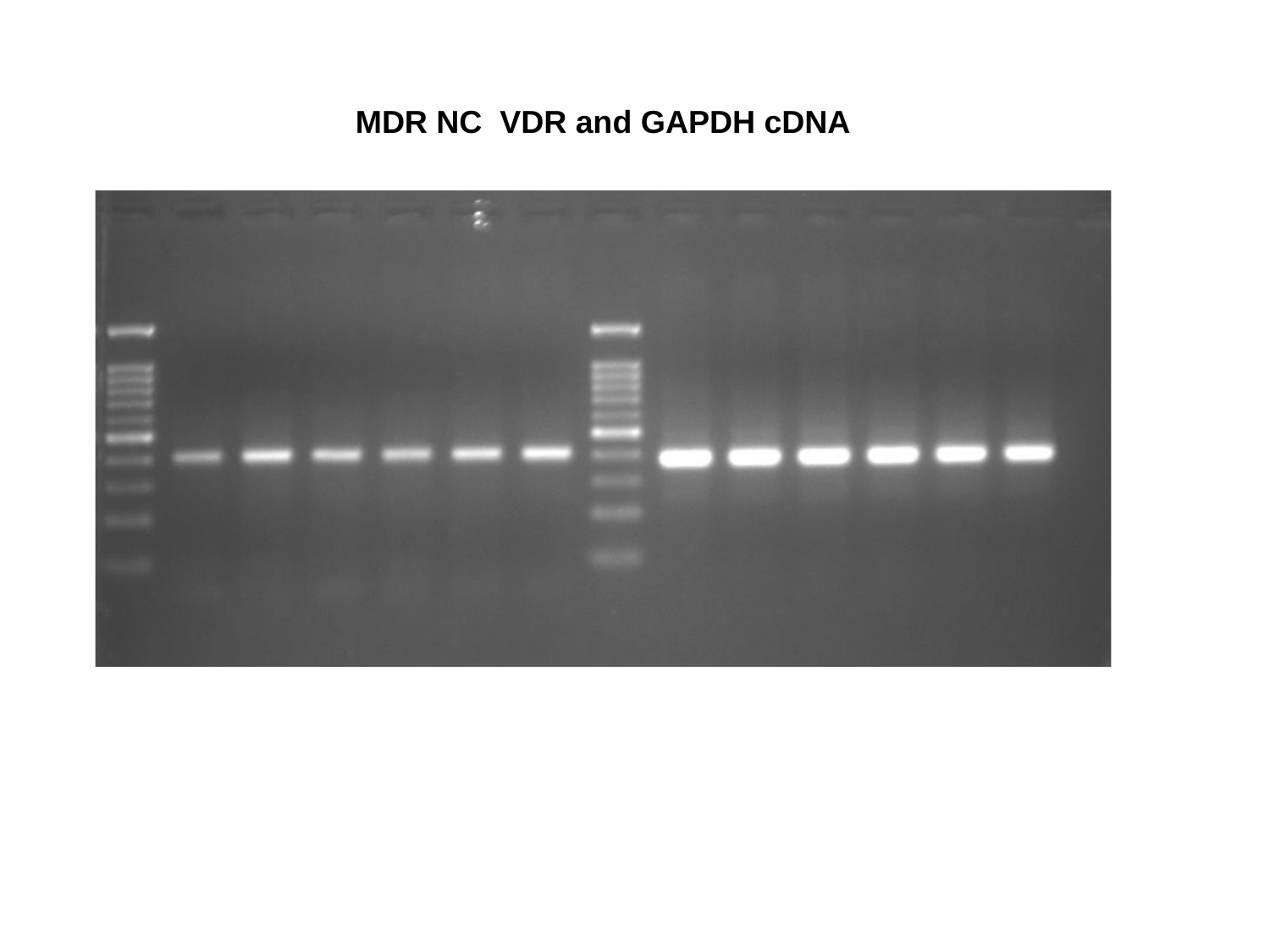

MDR NC VDR and GAPDH cDNA
