# Supplementary material for: Association of vitamin D receptor mRNA expression, vitamin D deficiency and genetic variant in patients with multi-drug resistant pulmonary tuberculosis
Source: BMC Infect Dis. 2025 Oct 15;25:1334. doi: 10.1186/s12879-025-11707-7 (PMC12522335; doi:10.1186/s12879-025-11707-7)
Supplement: Supplementary file 2 — Supplementary Material 2. [file 12879_2025_11707_MOESM2_ESM.docx]

**Serum vitamin D extraction procedure**

1. The glass tube for each standard, control and patient samples were labelled.

2. 500 μl of acetonitrile was added.

3. 50 μl of standard, control and patient samples were added slowly to acetonitrile.

4. The sample was vortexed and centrifuged using 1200 x g for 10 min at 20-25oC at 2700 rpm for 10 seconds.

5. 25 μl from each supernatant aliquots in duplicate were pipetted into separate appropriate labelled tubes, care was taken not to disturb the pellets.

**Procedure**

1. Total counts: 50 μl tracer + 1ml NSB/additional buffer.

2. NSB: 25 μl of 0 standard extracted + 50 μl tracer + 1ml NSB/additional buffer.

3. 25 μl of standard control and unknown samples extracted + 50 μl tracer +1ml antiserum were mixed.

4. Vortexed and incubated for 1-2hrs at 20-25oC.

5. 500 μl DAG precipitate complex were added and mixed thoroughly before and during use of all the tubes except control tubes.

6. After mixing incubated the tubes for 20-25 oC.

7. 500 μl NSB/additional buffer of all the tubes were added except control tubes and mixed the tubes well.

8. All the tubes were centrifuged for 20 minutes at 20-25oC at 1800xg at 3300 rpm except control tubes.

9. The supernatant was decanted except control tubes.

10. Radioactivity was counted for 1 minute.
