# Supplementary material for: Association of vitamin D receptor mRNA expression, vitamin D deficiency and genetic variant in patients with multi-drug resistant pulmonary tuberculosis
Source: BMC Infect Dis. 2025 Oct 15;25:1334. doi: 10.1186/s12879-025-11707-7 (PMC12522335; doi:10.1186/s12879-025-11707-7)
Supplement: Supplementary file 4 — Supplementary Material 4. [file 12879_2025_11707_MOESM4_ESM.doc]

**Title:** Association of vitamin D receptor mRNA expression, vitamin D deficiency and genetic variant in patients with multi-drug resistant pulmonary tuberculosis

**Authors:**

Jaishriram Rathored^1&2*^,

Surendra Kumar Sharma^1^,

V Sreenivas3,

Abhay Krishna Srivastava^4^

^1^Department of Medicine, All India Institute of Medical Sciences, New Delhi, India

^2^Central Research laboratory (CRL) and Molecular Diagnostics, School of Allied Health Sciences, Datta Meghe Institute of Higher Education and Research (DU), Sawangi (Meghe), Wardha, Maharashtra, India

^3^Department of Biostatistics, All India Institute of Medical Sciences, New Delhi, India

^4^Department of Laboratory Medicine, All India Institute of Medical Sciences, New Delhi, India

***Corresponding Author:**

Dr. Jaishriram Rathored^1&2*^,

Associate Professor

In-charge Central Research Laboratory and Molecular Diagnostics

Datta Meghe Institute of Higher Education and Research,

Sawangi (Meghe), Wardha- 442107, Maharashtra, India

Mobile: +91 8319380539

Email: jaishriz@gmail.com

**Abstract**

**Background** multi-drug resistant pulmonary tuberculosis (MDR-TB) is a serious global threat. Vitamin D acts as a ligand for the vitamin D receptor (VDR), a ligand-dependent transcription factor, for VDR mRNA expression. Serum vitamin D levels have not been investigated in MDR-TB.

**Objective** To study the association of mRNA expression with low vitamin D levels and VDR polymorphisms in patients with MDR-TB compared to normal controls.

**Methods** Study groups consisted of sputum smears and culture-positive MDR-TB at two hospitals in New Delhi, and normal controls were enrolled from a North Indian population. 100 individuals were consecutively enrolled in two groups (50 MDR-TB subjects and 50 controls). VDR mRNA expression in peripheral blood mononuclear cells (PBMC) was analysed by Real-time PCR. Serum 25-hydroxyvitamin D, intact parathyroid hormone (iPTH), and calcium (ionized and total) levels were measured, and the correlation between variables was determined. The association between VDR genotype and VDR mRNA expression was studied between MDR-TB and normal controls together with the genotypic and allelic frequencies of the *FokI, BsmI*, and *TaqI* VDR polymorphisms were also assessed in between the two groups.

**Results** A total of 100 subjects were recruited into two groups. Significantly low PBMC VDR mRNA, serum vitamin D, and calcium levels (ionized and total) were observed in MDR-TB patients. A significant positive correlation between VDR mRNA levels and serum vitamin D and calcium levels was found in patients with MDR-TB compared with normal controls. Similarly, VDR mRNA expression was 0.6-fold lower in MDR-TB patients than that in normal controls. VDR mRNA levels were significantly associated with *FokI* genotype in MDR-TB patients, whereas low levels of VDR mRNA were associated with the mutant *ff* genotype in both study groups. However, no significant association were found in genotypic and allelic frequency in the study groups

**Conclusion** In the present study, novel and substantial associations between VDR mRNA and serum vitamin D levels in MDR-TB patients have been identified. Decreased VDR expression levels may lead to impaired VDR signalling, which may raise inflammation in MDR-TB. This could be a reflection of variations in the actions of ligand-activated receptors across different tissues in patients and controls.

**Key words:** Vitamin D receptor, vitamin D mRNA expression, intact parathyroid hormone, multi-drug-resistant pulmonary tuberculosis, calcium levels.

**Introduction**

Tuberculosis (TB) is an infectious disease and a major health concern worldwide, with an estimated one-third of the world's population being infected with *Mycobacterium tuberculosis* including approximately 2 million people in India, with a prevalence of approximately 14 million (1). It is one of the main causes of global morbidity and mortality, resulting in approximately 1.7 million deaths annually, approximately 0.4 million of which are people being co-infected with human immunodeficiency virus (HIV) (1,2).

Pulmonary tuberculosis (PTB), the most common clinical form of the disease, is a granulomatous disease of the lungs caused by *Mycobacterium tuberculosis* (3). However, only 5-10% of infected individuals develop the disease (4). Furthermore, the management of tuberculosis has already faced significant risks due to the rise of multi-drug resistant (MDR) strains (5). The issue has been made more complex by the recent appearance of exceptionally drug-resistant (XDR) strains. With almost 410 000 new cases reported globally in 2022(1), multi-drug resistant (MDR) TB, in which resistance to at least the two most effective first-line treatments (rifampicin and isoniazid) (6,7), is becoming a more serious issue. About 100,000 new cases are reported each year, with China and India accounting for about half of these instances(1).

Vitamin D3 (1,25(OH)2D) is an immunoregulatory hormone that stimulates cell-mediated immunity by activating monocytes/macrophages (8,9). Parathyroid hormone (PTH) is the main hormone that regulates 1,25(OH)2D production via a negative feedback mechanism. Serum calcium homeostasis is maintained by the actions of PTH (which includes increased calcium reabsorption in the kidneys and bone) and (1,25(OH)2D) (increased calcium reabsorption in the kidneys and gut) (10). Vitamin D interacts with the vitamin D receptor (VDR) to mediate its effects. VDR is a nuclear receptor that controls the expression of several genes related to immune response, calcium homeostasis, and other biological processes when it binds with the active form of vitamin D (1,25-dihydroxyvitamin D, or calcitriol) (11). It has been suggested that mutant alleles of the VDR gene region may be associated with increased or decreased VDR mRNA expression (12), which in turn affects the protein function. The autocrine synthesis of 1,25(OH)2D is thought to have a significant role in controlling cell development and maturation, which lowers the likelihood that the cell may turn malignant. Additionally, 1-OHase in macrophages metabolizes 25(OH)D to 1,25(OH)2D. When LPS stimulates TLR2/1, the expression of the VDR and 1-OHase is increased (13). As a result, the expression of the VDR and 1-OHase increases. The macrophage's nuclear expression of cathelicidin (CD), a cationic peptide that destroys infectious organisms like Mycobacterium TB, rises in response to an increase in 1,25(OH)2D synthesis (12). Therefore, the vitamin D receptor gene is a good candidate for this study. The link between vitamin D and TB is an emerging field of research. Several studies have demonstrated vitamin D deficiency in patients with active TB by several studies (14–16). A number of studies have reported single nucleotide polymorphisms (SNP) in the vitamin D receptor (VDR) gene, which are thought to confer genetic differences in vitamin D physiology, susceptibility to active TB development (10,11,17–20), and TB treatment response (21,22).

To our knowledge, however, no studies have been published on the mRNA expression of VDR in patients with MDR-TB and variations in baseline vitamin D, calcium (ionized and total), and iPTH levels in MDR-TB patients and normal controls (23,24).

This study aimed to assess the correlation between VDR mRNA expression and vitamin D levels, and its association with VDR genotypes in MDR-TB.

**Subjects and Methods**

This prospective cross-sectional study was conducted at the All-India Institute of Medical Sciences (AIIMS) hospital (a tertiary centre) in New Delhi, India. The study recruited 26–29-year-old patients with MDR-TB and a control group of people from North India. The study was approved by the Institutional Ethical committee of All India Institute of Medical Sciences (IEC-AIIMS) Reference Number: A-08/5.5, New Delhi, India, and written informed consent was obtained from all subjects, including patients and normal controls.

**Multi-drug-resistant pulmonary tuberculosis Patients**

In the study, there were 50 patients and 50 controls. The diagnosis of MDR-TB was confirmed by a positive sputum smear (Ziehl-Neelsen method), culture (Lowenstein-Jensen medium) and conventional drug susceptibility testing (25–27). Inclusion criteria of the study includes Patients of either gender aged between 18 to 65 yrs. newly or old diagnosed MDR-TB patients (in vitro demonstration of resistance of Mycobacterium tuberculosis to rifampicin and isoniazid). Culture and sensitivity was done at the TB laboratory of New Delhi Tuberculosis Centre (NDTB) and Patients willing to give their written informed consent. Exclusion criteria were as follows: patients with category II or category III treatment according to the Indian Revised National Tuberculosis Control Programme (RNTCP) guidelines (28); presence of secondary immunodeficiency (29), for example, corticosteroid or other immunosuppressant drug use, diabetes mellitus, malignancy, co-infection with HIV, hepatitis B or hepatitis C virus, extrapulmonary TB in the absence of pulmonary involvement, concurrent cytotoxic chemotherapy, pregnancy or lactation, current or recent (<1year) use of vitamin D and/or calcium supplements; and patients with known seizure disorder (30).

**Normal Controls**

In this study, healthy volunteers (referred to as normal controls, n = 50) were randomly recruited from the general population with similar socioeconomic status and ethnic background. Subjects who had normal chest radiographs, serum biochemistry, liver function, complete blood count, and a body mass index (BMI) of 19 or over were included. Subjects with chronic illness, history of alcohol or drug abuse, family or personal history of tuberculosis, symptoms suggestive of malabsorption, and pregnant or breastfeeding mothers were excluded (31). Only individuals who had negative tuberculin skin test (TST) results were enrolled to exclude the possibility of latent tuberculosis infection. Subjects who had taken any vitamin D or calcium supplements within one year before recruitment were excluded from the control group. All individuals provided informed consent and were screened as negative for HIV infection.

**Estimation of Vitamin D, iPTH and calcium levels**

Blood samples were drawn without venostasis from the study subjects after overnight fasting. The serum was separated in a refrigerated centrifuge at 2500 × *g* for 5 min at 4°C and stored at -80°C in multiple aliquots until analysis. Serum 25-hydroxyvitamin D concentrations were estimated using the Diasorin® 25-hydroxyvitamin D Radio Immuno Assay (RIA) (normal range:9–37.6 ng/mL), which involves a two-step procedure. The first step involves the extraction of 25-hydroxyvitamin D from serum with acetonitrile, followed by processing according to the manufacturer’s instructions (32). Serum iPTH levels were measured using radioimmunoassay (RIA; Diasorin®, Stillwater, MN; normal range:13–54 pg/mL; intra-assay and inter-assay CVs:4% and 8%, respectively).

Serum ionized calcium and serum total calcium levels were measured using commercial kits (Roche, Mannheim, Germany) on a semi-automated analyzer (Hitachi Photometer 4020, Boehringer, Mannheim, Germany).

**VDR mRNA expression**

The vitamin D Receptor (VDR) mRNA and glyceraldehyde-3-phosphate dehydrogenase (GAPDH) was assessed by real-time PCR using gene-specific primers (VDR: sense, 5-gacatcggcatgatgaagg-3 ‘and antisense,5’ ctagggtcacagaagggtcatc-3’ and GAPDH: sense, 5’-ccaaggtcatccatgacaactttggt-3’ and antisense, 5’-tgttgaagtcagaggagaccacctg-3’). Total RNA was extracted from peripheral blood mononuclear cells (PBMC) (separated by Ficoll) using RNA-binding columns (Eppendorf-AG, Germany), and 2 μg was reverse transcribed using ImProm-II Reverse Transcription System (Promega, USA) in a 20-μl reaction (25 ^O^C for 5 min and extension at 42 ^O^C for 1 h). The VDR mRNA copy numbers were measured in relation to GAPDH mRNA by amplifying VDR and GAPDH cDNA in separate tubes using iQ^TM^ SYBR® Green Supermix (Bio-Rad, USA), and fluorescence signals were captured on an RT-PCR machine (iQ^TM^ 5 Cycler, Biorad,USA ). Reactions were performed in duplicate, and the RT-PCR conditions were as follows: initial denaturation at 95 ^O^C for 10 min, followed by 40 cycles of 95 ^O^C for 15 s, 60 ^O^C for 30 s, and 72 ^O^C for 10 min. The specificity of the amplified products was checked using post-PCR melting curve analysis and agarose gel electrophoresis (403 bp for VDR and 381 bp for GAPDH) **(Figure 1).**


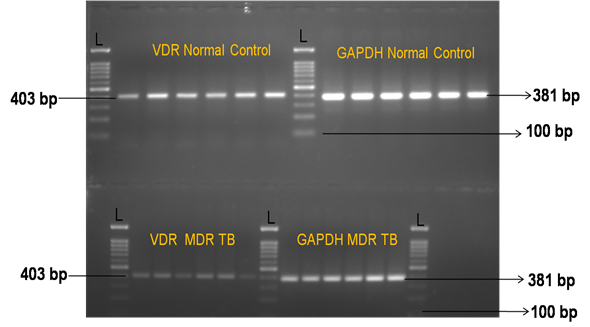


**Figure 1** Representative gel picture of vitamin D Receptor and GAPDH Real-Time PCR products

**Genotyping for the *VDR* gene Polymorphisms (*Bsm*I, *Fok*I, and *Taq*I)**

Genomic DNA was extracted from peripheral blood leukocytes using the DNA Blood Maxi Kit (Qiagen, Hilden, Germany). VDR genotypes at *the Bsm*I, *Taq*I, and *Fok*I SNP sites were assessed using PCR-RFLP analysis with a thermocycler (Eppendorf AG 22331, Hamburg, Germany) for PCR amplification. The polyacrylamide gel electrophoresis (PAGE)-purified primers (Bio Basic Inc., East Markham, Canada), DNA *Taq* polymerase, deoxynucleoside triphosphates, and various restriction endonucleases (Fermantas Inc., Hanover, MD) used in the study were commercially procured. The primer sequences, PCR cycling conditions, and PCR products were digested and processed as described by Vupputuri et al. (32). The presence of a restriction site was denoted by lower case letters (b, f, and t, for *Bsm*I, *Fok*I and *Taq*I) and absence by upper case letters (B, F, and T, for *Bsm*I, *Fok*I and *Taq*I).

**Statistical analysis**

Sample size was calculated considering mean Serum 25(OH)D, nmol/l 13.5 Standard Deviation 10.0 in MDR-TB while mean 34.9 Standard Deviation of 26.2 for Healthy control group with desired power of 0.99 and alpha error of 0.01 the minimum sample size required as 42 per group Data are presented as mean ± standard deviation (SD). Chi-square (ϰ2) test, two-sample t-test with equal variance, or one-way analysis of variance (ANOVA) followed by post hoc Bonferroni test were used to compare the differences between the study groups. Pearson’s correlation coefficient was estimated between VDR mRNA expression levels in MDR-TB patients and normal controls. We also perform a normality Kalmogorov smirnov test before conducting ANOVA and p value found greater than 0.05 is statically significant .All analysis was performed using STATA version 11.0 (Stata Corporation, College Station, TX, USA obtained/purchased a copyright license and were done by Professor of Statistics). A two-sided p-value of less than 0.05 was considered statistically significant.

**Results**

Clinical characteristics of MDR-TB patients (n=50) and a comparison of demographic characteristics with normal controls (n=50) were shown in (**Table 1).** A significantly lower BMI (p<0.001) was observed in patients with MDR-TB

|  | MDR-TB patients  (n=50)  n (%) | Normal controls (n=50) |
| --- | --- | --- |
| Age (yrs)  Sex M: F  BMI (kg/m^2^) | 27.5 ± 8.6  33:17  16.3 ± 2.4 | 29.6 ± 8.5  40:10  23.0 ± 2.1 |
| **Bacillary load**  3+  2+  1+ | 10 (20)  20 (40)  20 (40) | NA |
| **Culture status**  Positive  Negative | 50 (100)  0 (00) | NA |
| **Radiographic severity**  Unilateral  Bilateral  Minimal  Moderately advanced  Far advanced  **Number of Cavities**  No cavity  1  2  3  Multiple cavities | 1 (2)  49 (98)  01 (2)  09 (18)  40 (80)      01 (2)  07 (14)  08 (16)  06 (12)  28 (56) | NA  NA  NA |

**Table 1**: Baseline clinical characteristics of patients with MDR-TB and normal controls;

BMI indicates body mass index (kg/m^2^); age & BMI values are in mean ± SD;M=male,

F=female, NA=not applicable, MDR-TB, multidrug resistant pulmonary tuberculosis

**The details of the various biochemical parameters of the two study groups are shown in Table 2. Significantly lower mean serum 25-hydroxyvitamin D concentrations (5.9 ± 5.6 ng/mL; 13.3 ± 8.1 ng/mL; p<0.001) were found in MDR-TB. VDR mRNA expression was 0.6-fold less in MDR-TB patients than in normal controls (87.1 ± 39.5 copy number/10^6^ GAPDH copies; 160.0 ± 57.9 copy number/10^6^ GAPDH copies; p<0.001) (Table 2 and a significantly positive correlation between serum vitamin D and VDR mRNA levels (r=0.6; p<0.001 and r=0.4; p<0.001) was observed in patients with MDR-TB and normal controls** **(Figure 2), furthermore. Serum vitamin D was inversely correlated with mean serum iPTH concentration (41.0 ± 22.9 pg/mL;35.6 ± 20.3; p<0.001) (r= -0.3; p=0.02 and r= -0.4; p= 0.002) between the two study groups (Figure 3). Similarly, significantly lower mean serum ionised (4.0 ±0.5 mg/dL; 3.6 ± 0.7 mg/dL; p=0.006) and total calcium concentrations (8.0 ± 1.1 mg/dL; 7.3 ± 1.5 mg/dL; p=0.006; Table 2), and a significantly positive correlation between serum vitamin D and serum calcium levels (r=0.8; p<0.001 and r=0.8; p<0.001) were observed in patients with MDR-TB and normal controls (Figure 4). Likewise, low serum total protein (6.3 ± 0.9 g/dL; 8.4 ± 0.6; p<0.001) and serum albumin protein (3.4± 0.5 g/dL; 3.7 ± 0.6; p<0.02) were observed in patients with MDR-TB as compared to normal controls Table 2.**

| **Biochemical parameters** | **MDR-TB (n=50)**  **(mean ± SD)** | **Normal controls (n=50)**  **(mean ± SD)** | **p-Value** |
| --- | --- | --- | --- |
| **Serum total protein (g/dL)** | 6.3 ± 0.9 | 8.4 ± 0.6 | <0.001 |
| **Serum albumin (g/dL)** | 3.4 ± 0.5 | 3.7 ± 0.6 | 0.002 |
| **Serum Ionized calcium (mg/dL)** | 3.6 ± 0.7 | 4.0 ± 0.5 | <0.006 |
| **Serum calcium (mg/dL)** | 7.3 ± 1.5 | 8.0 ± 1.1 | <0.006 |
| **Corrected serum calcium (mg/dL) *** | 8.1 ± 1.6 | 8.4 ± 1.2 | 0.1 |
| **Serum iPTH (pg/mL)** | 41.0 ± 22.9 | 35.6 ± 20.3 | 0.8 |
| **Serum 25(OH)D (ng/mL)** | 5.9 ± 5.6 | 13.3 ± 8.7 | <0.001 |
| **VDR mRNA copies in PBMC/10^6^ GAPDH mRNA copies** | 87.1 ± 39.5 | 160 ± 57.9 | <0.001 |

**Table 2:** Baseline biochemical parameters of patients with MDR-TB and normal controls; MDR-TB=multi-drug resistant pulmonary tuberculosis, SD= standard deviation, iPTH, intact parathyroid hormone. *Corrected calcium (mg/dL)= measured total calcium (mg/dL) + 0.8 (4.4- serum albumin (g/dL), where 4.4 represent the average albumin level, PBMC= Peripheral blood mono nuclear cells**.**


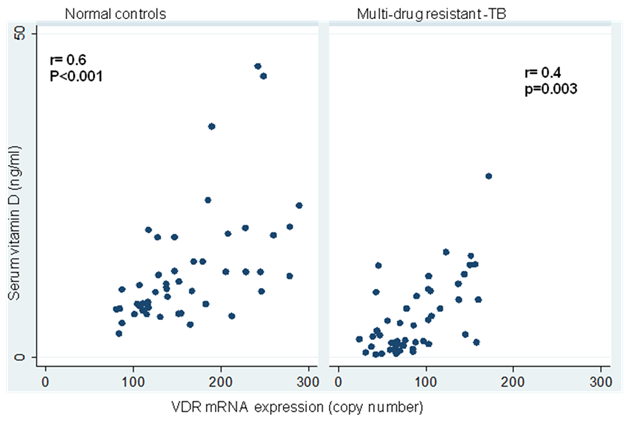


**Figure 2** Scatter plot showing correlation between serum vitamin D and mRNA levels in patients with MDR-TB and normal controls


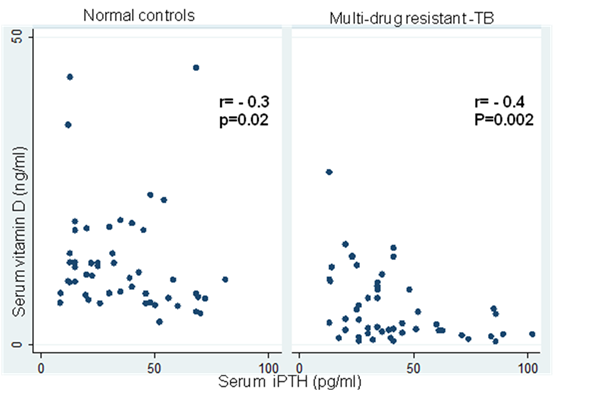


Figure 3 Scatter plot showing correlation between serum vitamin D and serum iPTH levels in patients with MDR-TB and normal controls


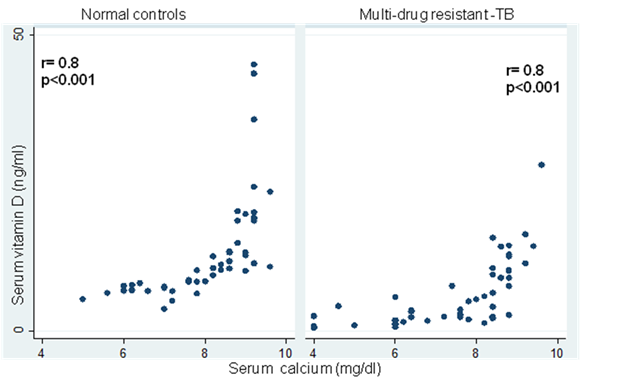


**Figure 4** Scatter plot showing correlation between serum vitamin D and serum calcium levels in patients with MDR-TB and normal controls

|  | Genotype polymorphism | p value  Global | p- value | | |
| --- | --- | --- | --- | --- | --- |
| *BSM I*  MDR-TB (n=50)  Normal Control (n=50) | *BB Bb bb*  95.3 ± 44.8 84.5 ± 26.3 73.6 ± 39.5  151.4 ± 51.3 156.2 ± 57.6 178.4 ± 66.6 | 0.13  0.60 | BB vs Bb  0.99  0.99 | BB vs bb  0.99  0.37 | Bb vs bb  0.99  0.99 |
| *Taq I*  MDR-TB (n=50)  Normal Control (n=50) | *TT*  *Tt*  *tt*  84.6 ± 37.8 90.3 ± 43.7 87.7 ± 39.1  162.0 ± 56.3 155. 8 ± 61.4 163.1 ± 62.4 | 0. 81  0.90 | TT vs Tt  0.99  0.99 | TT vs tt  0.99  0.99 | Tt vs tt  0.99  0.99 |
| *Fok I*  MDR-TB (n=50)  Normal Control (n=50) | *FF*  *Ff*  *ff*  100.0 ± 39.0 88.4 ± 36.3 36.0 ± 8.8  156.5 ± 51.2 170.6 ± 66.3 80.1 | 0.02  0.61 | FF vs Ff  0.88  0.99 | FF vs ff  0.003  0.59 | Ff vs ff  0.01  0.39 |

VDR mRNA levels were significantly associated with *FokI* VDR genotype in patients with MDR-TB (p=0.02 global), FF and ff genotype (p=0.003), and *Ff* and *ff* genotypes (p=0.01) (**Table 3**). However, no significant association of the VDR genotype (*Bsm*I*, Taq*I and *Fok*I) with mRNA levels was found in the normal control group. Although Table 4 represents the genotypic and allelic frequency distribution of VDR gene (Taq1, Fok1, and Bsm1) polymorphisms in MDRTB and healthy control, no statistically significant association was found between the two groups.

**Table 3** Association of mRNA levels with VDR genotype in patients with MDR-TB and normal controls; mRNA levels are shown in mean ± SD, MDR-TB= multi-drug resistant pulmonary tuberculosis

**Table 4** Genotypic and allelic frequency distribution of VDR gene (*Taq1, Fok1 and Bsm1*) polymorphism in MDRTB and Healthy control

| VDR Polymorphism Genotype | Healthy Controls (n=50) n% | MDR-TB (n=50) n% | OR (95% CI) | P Value* |
| --- | --- | --- | --- | --- |
| Bsml Genotypic Frequency |  |  |  |  |
| BB | 12 (24) | 17 (34) | 0.61 (0.26-1.47) | 0.27 |
| Bb | 27 (54) | 20 (40) | 1.76 (0.80-3.89) | 0.16 |
| bb | 11 (22) | 13 (26) | 0.8 (0.32-2.02) | 0.63 |
| Bsml Allelic Frequency ‡ |  |  |  |  |
| B | 26 (51) | 27 (54) | 0.92 (0.42-2.02) | 0.84 |
| b | 24 (49) | 23 (46) | 1.08 (0.49-2.38) | 0.84 |
| Taql Genotypic Frequency |  |  |  |  |
| TT | 24 (48) | 21 (42) | 1.27 (0.58-2.81) | 0.54 |
| Tt | 20 (40) | 20 (40) | 1 (0.45-2.23) | 1.0 |
| tt | 6 (12) | 9 (18) | 0.62 (0.2-1.9)) | 0.4 |
| Taql Allelic Frequency ‡ |  |  |  |  |
| T | 34 (68) | 31 (62) | 1.3 (0.57-2.97) | 0.52 |
| t | 16 (32) | 19 (38) | 0.77 (0.34-1.75) | 0.52 |
| Fokl Genotypic Frequency |  |  |  |  |
| FF | 29 (58) | 21 (42) | 1.91 (0.86-4.22) | 0.10 |
| Ff | 19 (38) | 25 (50) | 0.61 (0.25-1.36) | 0.22 |
| ff | 2 (4) | 4 (8) | 0.48 (0.08-2.74) | 0.49 |
| Fokl Allelic Frequency ‡ |  |  |  |  |
| F | 39 (78) | 34 (68) | 0.63 (0.21-1.87) | 0.26 |
| f | 11 (22) | 16 (32) | 0.6 (0.24-1.47) | 0.26 |

* P < 0.05 was considered statistically significant, ‡ Allelic frequency is double the genotypic frequency (2n). VDR = vitamin D receptor; MDR-TB = multidrug-resistant tuberculosis; OR = odds ratio; CI = confidence interval

**Discussion**

To our knowledge, this is the first study to explore VDR mRNA expression and its association with vitamin D levels and VDR genotypes in patients with MDR-TB compared to normal controls.

The finding from the current study, MDR-TB patients had considerably lower mean serum 25-hydroxyvitamin D concentrations, and their VDR mRNA expression was 0.6 times lower than that of normal controls. Additionally, we observed that the FokI VDR genotype in MDR-TB patients had a strong association with their VDR mRNA levels. The increased rate of MDR-TB coincided with the development of vitamin D deficiency, probably due to malnutrition and decreased sunlight exposure. A meta-analysis of the association between low vitamin D levels and active tuberculosis reported that low serum vitamin D levels are associated with an increased risk of active tuberculosis (14). Previous studies associated with an increased risk of TB and vitamin D deficiency have only investigated vitamin D levels only (10,15,16). In this study, serum calcium (ionized and total) and intact PTH levels were performed on both subjects to rule out these as confounding factors, and a significantly positive and negative correlation was found with vitamin D levels, respectively, which was as expected in patients without disorders affecting calcium homeostasis.

The VDR gene, located on chromosome 12q, has 14 exons, of which 6 are present in the 5’ untranslated region (33). VDR, which is a member of the nuclear hormone receptor superfamily, is a ligand-dependent transcription factor. The 1,25-(OH)2 D3 ligand interacts with VDR and mediates its biological function by inducing conformational changes that promote its heterodimerization with the Retinoid X Receptor (RXR), followed by translocation of the complex (RXR-VDR) into the nucleus. The RXR-VDR heterodimer regulates the function of 1,25- (OH)2 D3 by binding to vitamin D3 responsive elements (VDRE) in the promoter regions of the gene. By specifically targeting inducible transcription factors like NFAT in IL-2, 1,25-(OH)2 D3 may regulate the expression of certain genes, such as cytokine genes, in the absence of conventional responsive elements (33). By binding VDRE (Vitamin D Responsive elements) in the target gene's promoter region, both liganded and unliganded VDR can directly control gene transcription. It can also occupy recognition sites of other transcription factors, including NFAT, NF-̙B, and AP-1, and disrupt their signaling pathways (34). It has been shown that 1,25-(OH)2 D3 has an inhibitory effect on the overall Th1 response by repressing the transcription of IL-2, IFN-c, GM-CSF, and IL-12 and upregulating the production of Th2 cytokines IL-4 and (TGF-β1) (33), and thus has an immunomodulatory role. It also enhances the differentiation of TH2 cells from naive CD4 cells. Additionally, 1,25-(OH)2 D3 modulates the expression of HLA class II alleles in monocytes and human bone cells (33).

In the present study, we found that the *FokI* polymorphism was significantly associated with VDR mRNA levels. It has been shown that, the VDR is expressed in large number of tissues, it is not surprising that ligand-activated VDR modulates the expression of many genes (33). The *FokI* polymorphism results in the incorporation of three extra amino acids in the NH2 terminus of the VDR protein, which influences transcriptional activity by modulating the interaction with transcription factor IIB (TFIIB) (35,36). Alternatively, the *FokI* polymorphism interferes with the formation of VDR heterodimers with the retinoid X receptor, forming effector complexes (37). However, no such events have been reported for *BsmI* and the *TaqI* polymorphisms, which are located between exons 8 and 9 and exon 9, respectively. It has been reported that the *FokI* genotype contributes to feedback control of the expression of the 25-hydroxyvitamin D-1—hydroxylase (CYP27B1) gene, which is located in the same chromosomal region as the VDR gene and is active in PBMCs (38,39). Vitamin D deficiency leads to an increase in PBMC production by activated vitamin D (40). In accordance with the much lower serum calcium and vitamin D levels, MDR-TB patients exhibited a significantly higher mean serum iPTH content than the other two groups, as reported by Rathored et al. (2023). This implies that the outcomes are not the consequence of an endocrine problem because it appears to represent typical physiological reactions. PTB Cat I patients had a greater sun exposure index (SEI) than the MDR-TB and healthy control groups, which were comparable. Although the higher exposure in PTB Cat I patients compared to healthy controls may cast doubt on this association, this often showed a positive correlation with serum 25(OH)D levels, suggesting, as other studies have shown, that food may be a stronger predictor of the lower serum levels in PTB Cat I (41).

Interestingly, we observed a significant variation in VDR mRNA levels with the *FokI* polymorphism. This study demonstrates that *FokI* genotype may contribute to the expression of VDR mRNA. In addition, homozygosity for the VDR *FokI f* genotype was associated with lower levels of PBMC VDR mRNA expressions in MDR-TB patients than in normal controls. This reflects differences in the effects of ligand-activated receptors between different tissues in patients and controls. We also found that serum vitamin D levels were directly correlated with PBMC VDR mRNA levels and serum calcium levels (total and ionized) in both groups (MDR-TB and normal controls), suggesting that VDR is a ligand-dependent transcription factor and that the ligand for VDR is Vitamin D3, that is, 1,25-(OH)_2_ D_3_ which mediates its biological actions through VDR.

Based to this study results, MDR-TB patients had 0.6 times lower VDR mRNA expression than normal controls. This could be the result of impaired VDR signalling, which could lead to elevated inflammation by causing inflammatory cytokines to be expressed more frequently. Our findings in MDR-TB patients are further supported by the fact that vitamin D supplementation improves immunity against tuberculosis by favourably modulating the production of cathelicidin (42). Remarkably, Selvaraj et al. reported lower levels of VDR protein and higher levels of plasma 1,25-(OH)2 D3 (42). Also, the study conducted by Rathored J et al. 2023 found a significant association in MDR-TB and DS-TB patients in context to dietary profile with vitamin D levels and sunlight exposure (43).

The limitations of this study are the exclusion of drug-susceptible PTB patients and that MDR patients had invariably received anti-TB treatment at some point, in many cases, within a year of being diagnosed with MDR-TB. This may be a confounding factor because of the documented vitamin D-lowering effect of anti-TB drugs (14). However, it would be interesting to know whether patients become more susceptible to re-infection with MDR strains due to lowered vitamin D levels, and therefore, develop severe disease. This reasoning is plausible because the hypothesis that vitamin D deficiency leads to susceptibility to TB seems to be stronger than the inverse (14).

This study presents major new findings: a significant association between *FokI* VDR polymorphisms and low PBMC VDR mRNA levels in MDR-TB patients compared with normal controls. This adds to much recent work on the VDR gene, vitamin D, VDR mRNA levels and MDR-TB, and suggests that the *FokI* (especially mutant allele *f* and homozygous *ff*) and low serum vitamin D levels may be linked to low VDR mRNA levels and increased susceptibility to MDR-TB. This indicates a need for further study in this area, including confirmation of these results using a proteomics approach, and in vitro studies were incorporated into trials of vitamin D supplementation in MDR-TB cases.

**Abbreviation-** VDR- Vitamin D receptor, PBMC- Peripheral blood mono nuclear cells, MDR- Multi drug Resistant, VDRE-Vitamin D Responsive elements, Retinoid X Receptor- RXR, NFAT-Nuclear factor of activated T-cells

**Declarations**

**Ethics approval and consent to participate-** The study was approved by the Institutional Ethical committee of All India Institute of Medical Sciences (IEC-AIIMS) Reference Number: A-08/5.5, New Delhi, India, and written informed consent was obtained from all subjects, including patients and normal controls. Ethics committee (AIIMS-IEC) approved the procedure involved in the present research.

**Clinical Trial**: Not applicable.

**Consent for publication-** All authors agreed and give their consent for publication

**Availability of data and material-** The data that support the findings of this study are available from the corresponding author, [Dr Jaishriram Rathored], upon reasonable request.

**Competing interests-** None (The authors declare no competing interests)

**Funding-** The study was funded by the Department of Biotechnology, Ministry of Science and Technology, Government of India, New Delhi (GRANT NO BT/PR/7898/Med/14/1179)

**Authors' contributions** – **JR:** conceptualization, methodology, data collection, visualization, investigation and writing of original draft and editing; **SKS:** study supervision and review of the original draft and gave his expert advice during the study tenure. **VS:** helps in statistical analysis; **AKS:** helps in biochemical investigation and reviewing the original draft. All authors have reviewed and approved the final draft of the manuscript.

**Acknowledgements-** We are grateful to all study participants and the Department of Medicine staff at AIIMS, New Delhi, for making the study a success.

**REFERENCES**

1. Global Tuberculosis Report 2022 [Internet]. [cited 2025 Mar 12]. Available from: https://www.who.int/teams/global-tuberculosis-programme/tb-reports/global-tuberculosis-report-2022

2. Yin D, Zhong Y, Ling S, Lu S, Wang X, Jiang Z, et al. Dendritic-cell-targeting virus-like particles as potent mRNA vaccine carriers. Nat Biomed Eng. 2025 Feb;9(2):185–200.

3. Wang H, Li B, Sun Y, Ma Q, Feng Y, Jia Y, et al. NIR-II AIE Luminogen-Based Erythrocyte-Like Nanoparticles with Granuloma-Targeting and Self-Oxygenation Characteristics for Combined Phototherapy of Tuberculosis. Adv Mater. 2024 Sep;36(38):e2406143.

4. Sharma S, Rathored J, Ghosh B, Sharma SK. Genetic polymorphisms in TNF genes and tuberculosis in North Indians. BMC Infect Dis [Internet]. 2010 Jun 10 [cited 2025 Mar 12];10:165. Available from: https://www.ncbi.nlm.nih.gov/pmc/articles/PMC2894837/

5. Wang W, Wang J, Hu Z, Yan X, Gao Q, Li X, et al. Advancing Aggregation-Induced Emission-Derived Biomaterials in Viral, Tuberculosis, and Fungal Infectious Diseases. Aggregate [Internet]. [cited 2025 Mar 12];n/a(n/a):e715. Available from: https://onlinelibrary.wiley.com/doi/abs/10.1002/agt2.715

6. Lin X, Yu T, Zhang L, Chen S, Chen X, Liao Y, et al. Silencing Op18/stathmin by RNA Interference Promotes the Sensitivity of Nasopharyngeal Carcinoma Cells to Taxol and High-Grade Differentiation of Xenografted Tumours in Nude Mice. Basic Clin Pharmacol Toxicol. 2016 Dec;119(6):611–20.

7. Zhang X, Zhang Y, Xia W, Liu Y, Mao H, Bao L, et al. The relationship between vitamin D level and second acid-fast bacilli (AFB) smear-positive during treatment for TB patients was inferred by Bayesian network. PLoS One. 2022;17(5):e0267917.

8. Rathored J, Sharma SK, Banavaliker JN, Sreenivas V, Srivastava AK. Response to treatment and low serum vitamin D levels in North Indian patients with treatment-naive category I and multi-drug resistant pulmonary tuberculosis. Ann Med [Internet]. [cited 2025 Mar 12];56(1):2407066. Available from: https://www.ncbi.nlm.nih.gov/pmc/articles/PMC11421155/

9. Selvaraj P, Alagarasu K, Harishankar M, Vidyarani M, Narayanan PR. Regulatory region polymorphisms of vitamin D receptor gene in pulmonary tuberculosis patients and normal healthy subjects of south India. Int J Immunogenet. 2008 Jun;35(3):251–4.

10. Wilkinson RJ, Llewelyn M, Toossi Z, Patel P, Pasvol G, Lalvani A, et al. Influence of vitamin D deficiency and vitamin D receptor polymorphisms on tuberculosis among Gujarati Asians in west London: a case-control study. Lancet. 2000 Feb 19;355(9204):618–21.

11. Bornman L, Campbell SJ, Fielding K, Bah B, Sillah J, Gustafson P, et al. Vitamin D receptor polymorphisms and susceptibility to tuberculosis in West Africa: a case-control and family study. J Infect Dis. 2004 Nov 1;190(9):1631–41.

12. Morrison NA, Qi JC, Tokita A, Kelly PJ, Crofts L, Nguyen TV, et al. Prediction of bone density from vitamin D receptor alleles. Nature. 1994 Jan 20;367(6460):284–7.

13. Rathored J, Sharma SK, Singh B, Banavaliker JN, Sreenivas V, Srivastava AK, et al. Risk and outcome of multidrug-resistant tuberculosis: vitamin D receptor polymorphisms and serum 25(OH)D. Int J Tuberc Lung Dis. 2012 Nov;16(11):1522–8.

14. Nnoaham KE, Clarke A. Low serum vitamin D levels and tuberculosis: a systematic review and meta-analysis. Int J Epidemiol. 2008 Feb;37(1):113–9.

15. Davies PD, Brown RC, Woodhead JS. Serum concentrations of vitamin D metabolites in untreated tuberculosis. Thorax. 1985 Mar;40(3):187–90.

16. Sita-Lumsden A, Lapthorn G, Swaminathan R, Milburn HJ. Reactivation of tuberculosis and vitamin D deficiency: the contribution of diet and exposure to sunlight. Thorax. 2007 Nov;62(11):1003–7.

17. Lewis SJ, Baker I, Davey Smith G. Meta-analysis of vitamin D receptor polymorphisms and pulmonary tuberculosis risk. Int J Tuberc Lung Dis. 2005 Oct;9(10):1174–7.

18. Motsinger-Reif AA, Antas PRZ, Oki NO, Levy S, Holland SM, Sterling TR. Polymorphisms in IL-1beta, vitamin D receptor Fok1, and Toll-like receptor 2 are associated with extrapulmonary tuberculosis. BMC Med Genet. 2010 Mar 2;11:37.

19. Selvaraj P, Chandra G, Kurian SM, Reetha AM, Narayanan PR. Association of vitamin D receptor gene variants of BsmI, ApaI and FokI polymorphisms with susceptibility or resistance to pulmonary tuberculosis. CURRENT SCIENCE. 2003;84(12).

20. Lombard Z, Dalton DL, Venter PA, Williams RC, Bornman L. Association of HLA-DR, -DQ, and vitamin D receptor alleles and haplotypes with tuberculosis in the Venda of South Africa. Hum Immunol. 2006 Aug;67(8):643–54.

21. Babb C, van der Merwe L, Beyers N, Pheiffer C, Walzl G, Duncan K, et al. Vitamin D receptor gene polymorphisms and sputum conversion time in pulmonary tuberculosis patients. Tuberculosis (Edinb). 2007 Jul;87(4):295–302.

22. Roth DE, Soto G, Arenas F, Bautista CT, Ortiz J, Rodriguez R, et al. Association between vitamin D receptor gene polymorphisms and response to treatment of pulmonary tuberculosis. J Infect Dis. 2004 Sep 1;190(5):920–7.

23. Zhou Y, Li Q, Pan R, Wang Q, Zhu X, Yuan C, et al. Regulatory roles of three miRNAs on allergen mRNA expression in Tyrophagus putrescentiae. Allergy. 2022 Feb;77(2):469–82.

24. Zhang YW, Zheng XW, Liu YJ, Fang L, Pan ZF, Bao MH, et al. Effect of Oridonin on Cytochrome P450 Expression and Activities in HepaRG Cell. Pharmacology. 2018;101(5–6):246–54.

25. Pu X, Sheng S, Fu Y, Yang Y, Xu G. Construction of circRNA–miRNA–mRNA ceRNA regulatory network and screening of diagnostic targets for tuberculosis. Ann Med [Internet]. [cited 2025 Mar 12];56(1):2416604. Available from: https://www.ncbi.nlm.nih.gov/pmc/articles/PMC11497567/

26. Treatment of Tuberculosis: Guidelines [Internet]. 4th ed. Geneva: World Health Organization; 2010 [cited 2025 Mar 12]. (WHO Guidelines Approved by the Guidelines Review Committee). Available from: http://www.ncbi.nlm.nih.gov/books/NBK138748/

27. Laboratory services. In: Implementing the WHO Stop TB Strategy: A Handbook for National Tuberculosis Control Programmes [Internet]. World Health Organization; 2008 [cited 2025 Mar 12]. Available from: https://www.ncbi.nlm.nih.gov/books/NBK310753/

28. Khatri GR, Frieden TR. Controlling tuberculosis in India. N Engl J Med. 2002 Oct 31;347(18):1420–5.

29. Zhou Y, Li L, Yu Z, Gu X, Pan R, Li Q, et al. Dermatophagoides pteronyssinus allergen Der p 22: Cloning, expression, IgE-binding in asthmatic children, and immunogenicity. Pediatr Allergy Immunol. 2022 Aug;33(8):e13835.

30. Guo Z, Guan K, Bao M, He B, Lu J. LINC-PINT plays an anti-tumor role in nasopharyngeal carcinoma by binding to XRCC6 and affecting its function. Pathol Res Pract. 2024 Aug;260:155460.

31. Feng C, Wang Y, Xu J, Zheng Y, Zhou W, Wang Y, et al. Precisely Tailoring Molecular Structure of Doxorubicin Prodrugs to Enable Stable Nanoassembly, Rapid Activation, and Potent Antitumor Effect. Pharmaceutics. 2024 Dec 11;16(12):1582.

32. Vupputuri MR, Goswami R, Gupta N, Ray D, Tandon N, Kumar N. Prevalence and functional significance of 25-hydroxyvitamin D deficiency and vitamin D receptor gene polymorphisms in Asian Indians. Am J Clin Nutr. 2006 Jun;83(6):1411–9.

33. Ogunkolade BW, Boucher BJ, Prahl JM, Bustin SA, Burrin JM, Noonan K, et al. Vitamin D receptor (VDR) mRNA and VDR protein levels in relation to vitamin D status, insulin secretory capacity, and VDR genotype in Bangladeshi Asians. Diabetes. 2002 Jul;51(7):2294–300.

34. van Etten E, Verlinden L, Giulietti A, Ramos-Lopez E, Branisteanu DD, Ferreira GB, et al. The vitamin D receptor gene FokI polymorphism: functional impact on the immune system. Eur J Immunol. 2007 Feb;37(2):395–405.

35. Arai H, Miyamoto K, Taketani Y, Yamamoto H, Iemori Y, Morita K, et al. A vitamin D receptor gene polymorphism in the translation initiation codon: effect on protein activity and relation to bone mineral density in Japanese women. J Bone Miner Res. 1997 Jun;12(6):915–21.

36. Jurutka PW, Remus LS, Whitfield GK, Thompson PD, Hsieh JC, Zitzer H, et al. The polymorphic N terminus in human vitamin D receptor isoforms influences transcriptional activity by modulating interaction with transcription factor IIB. Mol Endocrinol. 2000 Mar;14(3):401–20.

37. Haussler MR, Whitfield GK, Haussler CA, Hsieh JC, Thompson PD, Selznick SH, et al. The nuclear vitamin D receptor: biological and molecular regulatory properties revealed. J Bone Miner Res. 1998 Mar;13(3):325–49.

38. Smith SJ, Rucka AK, Berry JL, Davies M, Mylchreest S, Paterson CR, et al. Novel mutations in the 1alpha-hydroxylase (P450c1) gene in three families with pseudovitamin D-deficiency rickets resulting in loss of functional enzyme activity in blood-derived macrophages. J Bone Miner Res. 1999 May;14(5):730–9.

39. Bell NH. Renal and nonrenal 25-hydroxyvitamin D-1alpha-hydroxylases and their clinical significance. J Bone Miner Res. 1998 Mar;13(3):350–3.

40. Dusso AS, Finch J, Brown A, Ritter C, Delmez J, Schreiner G, et al. Extrarenal production of calcitriol in normal and uremic humans. J Clin Endocrinol Metab. 1991 Jan;72(1):157–64.

41. Goswami R, Mondal AM, Tomar N, Ray D, Chattopadhyay P, Gupta N, et al. Presence of 25(OH)D deficiency and its effect on vitamin D receptor mRNA expression. Eur J Clin Nutr. 2009 Mar;63(3):446–9.

42. Selvaraj P, Prabhu Anand S, Harishankar M, Alagarasu K. Plasma 1,25 dihydroxy vitamin D3 level and expression of vitamin d receptor and cathelicidin in pulmonary tuberculosis. J Clin Immunol. 2009 Jul;29(4):470–8.

43. Rathored J, Sharma SK, Chauhan A, Singh B, Banavaliker JN, Sreenivas V, et al. Low serum vitamin D in North Indian multi-drug resistant pulmonary tuberculosis patients: the role of diet and sunlight. Ann Med. 2023;55(2):2291554.
