# Supplementary material for: Association of vitamin D receptor mRNA expression, vitamin D deficiency and genetic variant in patients with multi-drug resistant pulmonary tuberculosis
Source: BMC Infect Dis. 2025 Oct 15;25:1334. doi: 10.1186/s12879-025-11707-7 (PMC12522335; doi:10.1186/s12879-025-11707-7)
Supplement: Supplementary file 7 — Supplementary Material 7. [file 12879_2025_11707_MOESM7_ESM.doc]

**Title:** Association of vitamin D receptor mRNA expression, vitamin D deficiency and genetic variant in patients with multi-drug resistant pulmonary tuberculosis

**Authors:**

Jaishriram Rathored^1&2*^,

Surendra Kumar Sharma^1^,

V Sreenivas3,

Abhay Krishna Srivastava^4^

^1^Department of Medicine, All India Institute of Medical Sciences, New Delhi, India

^2^Central Research laboratory (CRL) and Molecular Diagnostics, School of Allied Health Sciences, Datta Meghe Institute of Higher Education and Research (DU), Sawangi (Meghe), Wardha, Maharashtra, India

^3^Department of Biostatistics, All India Institute of Medical Sciences, New Delhi, India

^4^Department of Laboratory Medicine, All India Institute of Medical Sciences, New Delhi, India

***Corresponding Author:**

Dr. Jaishriram Rathored^1&2*^,

Associate Professor

In-charge Central Research Laboratory and Molecular Diagnostics

Datta Meghe Institute of Higher Education and Research,

Sawangi (Meghe), Wardha- 442107, Maharashtra, India

Mobile: +91 8319380539

Email: jaishriz@gmail.com

**Abstract**

**Background** Multi-drug resistant pulmonary tuberculosis (MDR-TB), is a serious threat to world health. Serum levels of vitamin D, a ligand for the VDR that controls VDR mRNA expression, are still poorly understood in MDR-TB.

**Objective** To study the association of mRNA expression with low vitamin D levels and VDR polymorphisms in patients with MDR-TB compared to normal controls.

**Methods** Study groups consisted of sputum smears and culture-positive MDR-TB at two hospitals in New Delhi, and normal controls were enrolled from a North Indian population. A total 100 (50 MDR-TB subjects and 50 controls) were consecutively enrolled. VDR mRNA expression in peripheral blood mononuclear cells (PBMC) was analysed by Real-time PCR. Serum 25-hydroxyvitamin D, intact parathyroid hormone (iPTH), and calcium (ionized and total) levels were measured, and the correlation between variables was determined. The association between VDR genotype and VDR mRNA expression was studied between MDR-TB and normal controls together with the genotypic and allelic frequencies of the *FokI, BsmI*, and *TaqI* VDR polymorphisms were also assessed in between the two groups.

**Results** To investigate the role of VDR gene expression and FokI polymorphism in MDR-TB, a total of 100 patients were split into two groups. VDR mRNA expression significantly decreased in MDR-TB patients, being 0.6 times lower than in healthy controls. Notably, the ff genotype was associated with reduced VDR expression, indicating a functional impact on gene regulation. However, there was no appreciable variation in the groups' distribution of FokI alleles and genotypes. These findings highlight the importance of merging genetic and expression data, showing that while the ff variation influences individual expression, it does not distinguish MDR-TB patients from controls.

**Conclusion** In present study, the VDR gene's FokI polymorphism affects the levels of VDR mRNA expression, with the ff genotype linked to lower expression in both MDR-TB patients and healthy individuals. Nonetheless, there were no appreciable differences in the genotypic and allelic frequencies of FokI between the groups. These findings suggest that the location of the FokI variation in the population may not be as important to MDR-TB susceptibility as its functional impact on gene expression.

**Key words:** Vitamin D receptor, vitamin D mRNA expression, intact parathyroid hormone, multi-drug-resistant pulmonary tuberculosis, calcium levels.

**Introduction**

Tuberculosis (TB) is an infectious disease and a major health concern worldwide, with an estimated one-third of the world's population being infected with *Mycobacterium tuberculosis* including approximately 2 million people in India, with a prevalence of approximately 14 million (1). It is one of the main causes of global morbidity and mortality, resulting in approximately 1.7 million deaths annually, approximately 0.4 million of which are people being co-infected with human immunodeficiency virus (HIV) (1,2).

Pulmonary tuberculosis (PTB), the most common clinical form of the disease, is a granulomatous disease of the lungs caused by *Mycobacterium tuberculosis* (3). However, only 5-10% of infected individuals develop the disease (4). Furthermore, the management of tuberculosis has already faced significant risks due to the rise of multi-drug resistant (MDR) strains (5). The issue has been made more complex by the recent appearance of exceptionally drug-resistant (XDR) strains. With almost 410 000 new cases reported globally in 2022(1), multi-drug resistant (MDR) TB, in which resistance to at least the two most effective first-line treatments (rifampicin and isoniazid) (6,7), is becoming a more serious issue. About 100,000 new cases are reported each year, with China and India accounting for about half of these instances(1).

Vitamin D3 (1,25(OH)2D) is an immunoregulatory hormone that stimulates cell-mediated immunity by activating monocytes/macrophages (8,9). Parathyroid hormone (PTH) is the main hormone that regulates 1,25(OH)2D production via a negative feedback mechanism. Serum calcium homeostasis is maintained by the actions of PTH (which includes increased calcium reabsorption in the kidneys and bone) and (1,25(OH)2D) (increased calcium reabsorption in the kidneys and gut) (10). Vitamin D interacts with the vitamin D receptor (VDR) to mediate its effects. VDR is a nuclear receptor that controls the expression of several genes related to immune response, calcium homeostasis, and other biological processes when it binds with the active form of vitamin D (1,25-dihydroxyvitamin D, or calcitriol) (11). It has been suggested that mutant alleles of the VDR gene region may be associated with increased or decreased VDR mRNA expression (12), which in turn affects the protein function. The autocrine synthesis of 1,25(OH)2D is thought to have a significant role in controlling cell development and maturation, which lowers the likelihood that the cell may turn malignant. Additionally, 1-OHase in macrophages metabolizes 25(OH)D to 1,25(OH)2D. When LPS stimulates TLR2/1, the expression of the VDR and 1-OHase is increased (13). As a result, the expression of the VDR and 1-OHase increases. The macrophage's nuclear expression of cathelicidin (CD), a cationic peptide that destroys infectious organisms like Mycobacterium TB, rises in response to an increase in 1,25(OH)2D synthesis (12). Therefore, the vitamin D receptor gene is a good candidate for this study. The link between vitamin D and TB is an emerging field of research. Several studies have demonstrated vitamin D deficiency in patients with active TB by several studies (14–16). A number of studies have reported single nucleotide polymorphisms (SNP) in the vitamin D receptor (VDR) gene, which are thought to confer genetic differences in vitamin D physiology, susceptibility to active TB development (10,11,17–20), and TB treatment response (21,22).

To our knowledge, however, no studies have been published on the mRNA expression of VDR in patients with MDR-TB and variations in baseline vitamin D, calcium (ionized and total), and iPTH levels in MDR-TB patients and normal controls (23,24).

This study aimed to assess the correlation between VDR mRNA expression and vitamin D levels, and its association with VDR genotypes in MDR-TB.

**Subjects and Methods**

This prospective cross-sectional study was conducted at the All-India Institute of Medical Sciences (AIIMS) hospital (a tertiary centre) in New Delhi, India. The study recruited 26–29-year-old patients with MDR-TB and a control group of people from North India. The study was approved by the Institutional Ethical committee of All India Institute of Medical Sciences (IEC-AIIMS) Reference Number: A-08/5.5, New Delhi, India, and written informed consent was obtained from all subjects, including patients and normal controls.

**Multi-drug-resistant pulmonary tuberculosis Patients**

In the study, there were 50 patients and 50 controls. The diagnosis of MDR-TB was confirmed by a positive sputum smear (Ziehl-Neelsen method), culture (Lowenstein-Jensen medium) and conventional drug susceptibility testing (25–27). Inclusion criteria of the study includes Patients of either gender aged between 18 to 65 yrs. newly or old diagnosed MDR-TB patients (in vitro demonstration of resistance of Mycobacterium tuberculosis to rifampicin and isoniazid). Culture and sensitivity was done at the TB laboratory of New Delhi Tuberculosis Centre (NDTB) and Patients willing to give their written informed consent. Exclusion criteria were as follows: patients with category II or category III treatment according to the Indian Revised National Tuberculosis Control Programme (RNTCP) guidelines (28); presence of secondary immunodeficiency (29), for example, corticosteroid or other immunosuppressant drug use, diabetes mellitus, malignancy, co-infection with HIV, hepatitis B or hepatitis C virus, extrapulmonary TB in the absence of pulmonary involvement, concurrent cytotoxic chemotherapy, pregnancy or lactation, current or recent (<1year) use of vitamin D and/or calcium supplements; and patients with known seizure disorder (30).

**Normal Controls**

In this study, healthy volunteers (referred to as normal controls, n = 50) were randomly recruited from the general population with similar socioeconomic status and ethnic background. Subjects who had normal chest radiographs, serum biochemistry, liver function, complete blood count, and a body mass index (BMI) of 19 or over were included. Subjects with chronic illness, history of alcohol or drug abuse, family or personal history of tuberculosis, symptoms suggestive of malabsorption, and pregnant or breastfeeding mothers were excluded (31). Only individuals who had negative tuberculin skin test (TST) results were enrolled to exclude the possibility of latent tuberculosis infection. Subjects who had taken any vitamin D or calcium supplements within one year before recruitment were excluded from the control group. All individuals provided informed consent and were screened as negative for HIV infection.

**Estimation of Vitamin D, iPTH and calcium levels**

Blood samples were drawn without venostasis from the study subjects after overnight fasting. The serum was separated in a refrigerated centrifuge at 2500 × *g* for 5 min at 4°C and stored at -80°C in multiple aliquots until analysis. Serum 25-hydroxyvitamin D concentrations were estimated using the Diasorin® 25-hydroxyvitamin D Radio Immuno Assay (RIA) (normal range:9–37.6 ng/mL), which involves a two-step procedure. The first step involves the extraction of 25-hydroxyvitamin D from serum with acetonitrile, followed by processing according to the manufacturer’s instructions (32). Serum iPTH levels were measured using radioimmunoassay (RIA; Diasorin®, Stillwater, MN; normal range:13–54 pg/mL; intra-assay and inter-assay CVs:4% and 8%, respectively).

Serum ionized calcium and serum total calcium levels were measured using commercial kits (Roche, Mannheim, Germany) on a semi-automated analyzer (Hitachi Photometer 4020, Boehringer, Mannheim, Germany).

**VDR mRNA expression**

Absolute quantification of VDR mRNA expression was performed using TaqMan-based qPCR. Standard curves were generated by serial dilution of synthetic VDR and GAPDH cDNA templates with known copy numbers. VDR mRNA copy numbers were normalized to 10⁶ copies of GAPDH mRNA. All reactions were run in triplicate, and only those with amplification efficiencies between 90–110% and R² >0.99 were included. No-template and negative reverse-transcription controls were included in each run to rule out contamination.

The vitamin D Receptor (VDR) mRNA and glyceraldehyde-3-phosphate dehydrogenase (GAPDH) was assessed by real-time PCR using gene-specific primers (VDR: sense, 5-gacatcggcatgatgaagg-3 ‘and antisense,5’ ctagggtcacagaagggtcatc-3’ and GAPDH: sense, 5’-ccaaggtcatccatgacaactttggt-3’ and antisense, 5’-tgttgaagtcagaggagaccacctg-3’). Total RNA was extracted from peripheral blood mononuclear cells (PBMC) (separated by Ficoll) using RNA-binding columns (Eppendorf-AG, Germany), and 2 μg was reverse transcribed using ImProm-II Reverse Transcription System (Promega, USA) in a 20-μl reaction (25 ^O^C for 5 min and extension at 42 ^O^C for 1 h). The VDR mRNA copy numbers were measured in relation to GAPDH mRNA by amplifying VDR and GAPDH cDNA in separate tubes using iQ^TM^ SYBR® Green Supermix (Bio-Rad, USA), and fluorescence signals were captured on an RT-PCR machine (iQ^TM^ 5 Cycler, Biorad,USA). Reactions were performed in duplicate, and the RT-PCR conditions were as follows: initial denaturation at 95 ^O^C for 10 min, followed by 40 cycles of 95 ^O^C for 15 s, 60 ^O^C for 30 s, and 72 ^O^C for 10 min. The specificity of the amplified products was checked using post-PCR melting curve analysis and agarose gel electrophoresis (403 bp for VDR and 381 bp for GAPDH) **(Figure 1).**


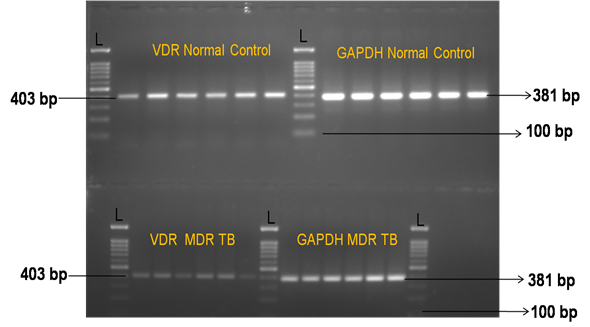


**Figure 1** Representative gel picture of vitamin D Receptor and GAPDH Real-Time PCR products

**Genotyping for the *VDR* gene Polymorphisms (*Bsm*I, *Fok*I, and *Taq*I)**

Genomic DNA was extracted from peripheral blood leukocytes using the DNA Blood Maxi Kit (Qiagen, Hilden, Germany). VDR genotypes at *the Bsm*I, *Taq*I, and *Fok*I SNP sites were assessed using PCR-RFLP analysis with a thermocycler (Eppendorf AG 22331, Hamburg, Germany) for PCR amplification. The polyacrylamide gel electrophoresis (PAGE)-purified primers (Bio Basic Inc., East Markham, Canada), DNA *Taq* polymerase, deoxynucleoside triphosphates, and various restriction endonucleases (Fermantas Inc., Hanover, MD) used in the study were commercially procured. The primer sequences, PCR cycling conditions, and PCR products were digested and processed as described by Vupputuri et al. (32). The presence of a restriction site was denoted by lower case letters (b, f, and t, for *Bsm*I, *Fok*I and *Taq*I) and absence by upper case letters (B, F, and T, for *Bsm*I, *Fok*I and *Taq*I). Four frequently explored single nucleotide polymorphisms (SNPs) are found in the VDR gene: ApaI (rs7975232), BsmI (rs1544410) (33), TaqI (rs731236) (33) and FokI (rs2228570) (34). These could all affect VDR function in different ways, such as receptor activity, translation efficiency, or mRNA stability. However ApaI (rs7975232) were not included in the present study.

**Statistical analysis**

Sample size was calculated considering mean Serum 25(OH)D, nmol/l 13.5 Standard Deviation 10.0 in MDR-TB while mean 34.9 Standard Deviation of 26.2 for Healthy control group with desired power of 0.99 and alpha error of 0.01 the minimum sample size required as 42 per group Data are presented as mean ± standard deviation (SD). Chi-square (ϰ2) test, two-sample t-test with equal variance, or one-way analysis of variance (ANOVA) followed by post hoc Bonferroni test were used to compare the differences between the study groups. **A multiple linear regression model was used to assess differences in serum 25(OH)D, total calcium, ionized calcium, and corrected calcium levels between MDR-TB patients and healthy controls, adjusting for age, sex, and BMI. The adjusted means and standard errors were calculated from the regression model estimates**. We also perform a normality Kalmogorov smirnov test before conducting ANOVA and p value found greater than 0.05 is statically significant .All analysis was performed using STATA version 11.0 (Stata Corporation, College Station, TX, USA obtained/purchased a copyright license and were done by Professor of Statistics). A two-sided p-value of less than 0.05 was considered statistically significant.

**Results**

Clinical characteristics of MDR-TB patients (n=50) and a comparison of demographic characteristics with normal controls (n=50) were shown in (**Table 1).** A significantly lower BMI (p<0.001) was observed in patients with MDR-TB

|  | MDR-TB patients  (n=50)  n (%) | Normal controls (n=50) |
| --- | --- | --- |
| Age (yrs)  Sex M: F  BMI (kg/m^2^) | 27.5 ± 8.6  33:17  16.3 ± 2.4 | 29.6 ± 8.5  40:10  23.0 ± 2.1 |
| **Bacillary load**  3+  2+  1+ | 10 (20)  20 (40)  20 (40) | NA |
| **Culture status**  Positive  Negative | 50 (100)  0 (00) | NA |
| **Radiographic severity**  Unilateral  Bilateral  Minimal  Moderately advanced  Far advanced  **Number of Cavities**  No cavity  1  2  3  Multiple cavities | 1 (2)  49 (98)  01 (2)  09 (18)  40 (80)      01 (2)  07 (14)  08 (16)  06 (12)  28 (56) | NA  NA  NA |

**Table 1**: Baseline clinical characteristics of patients with MDR-TB and normal controls;

BMI indicates body mass index (kg/m^2^); age & BMI values are in mean ± SD;M=male,

F=female, NA=not applicable, MDR-TB, multidrug resistant pulmonary tuberculosis

The details of the various biochemical parameters of the two study groups are shown in **Table 2**. Significantly lower mean serum 25-hydroxyvitamin D concentrations (5.9 ± 5.6 ng/mL; 13.3 ± 8.1 ng/mL; p<0.001) were found in MDR-TB. VDR mRNA expression was 0.6-fold less in MDR-TB patients than in normal controls (87.1 ± 39.5 copy number/10^6^ GAPDH copies; 160.0 ± 57.9 copy number/10^6^ GAPDH copies; p<0.001) **(Table 2** and a significantly positive correlation between serum vitamin D and VDR mRNA levels (r=0.6; p<0.001 and r=0.4; p<0.001) was observed in patients with MDR-TB and normal controls **(Figure 2),** furthermore. Serum vitamin D was inversely correlated with mean serum iPTH concentration (41.0 ± 22.9 pg/mL;35.6 ± 20.3; p<0.001) (r= -0.3; p=0.02 and r= -0.4; p= 0.002) between the two study groups Similarly, significantly lower mean serum ionised (4.0 ±0.5 mg/dL; 3.6 ± 0.7 mg/dL; p=0.006) and total calcium concentrations (8.0 ± 1.1 mg/dL; 7.3 ± 1.5 mg/dL; p=0.006; **Table 2**), and a significantly positive correlation between serum vitamin D and serum calcium levels (r=0.8; p<0.001 and r=0.8; p<0.001) were observed in patients with MDR-TB and normal controls (**Figure 3**). Likewise, low serum total protein (6.3 ± 0.9 g/dL; 8.4 ± 0.6; p<0.001) and serum albumin protein (3.4± 0.5 g/dL; 3.7 ± 0.6; p<0.02) were observed **in** patients with MDR-TB as compared to normal controls **Table 2.**

| **Biochemical parameters** | **MDR-TB (n=50)**  **(mean ± SD)** | **Normal controls (n=50)**  **(mean ± SD)** | **p-Value** |
| --- | --- | --- | --- |
| **Serum total protein (g/dL)** | 6.3 ± 0.9 | 8.4 ± 0.6 | <0.001 |
| **Serum albumin (g/dL)** | 3.4 ± 0.5 | 3.7 ± 0.6 | 0.002 |
| **Serum Ionized calcium (mg/dL)** | 3.6 ± 0.7 | 4.0 ± 0.5 | <0.006 |
| **Serum calcium (mg/dL)** | 7.3 ± 1.5 | 8.0 ± 1.1 | <0.006 |
| **Corrected serum calcium (mg/dL) *** | 8.1 ± 1.6 | 8.4 ± 1.2 | 0.1 |
| **Serum iPTH (pg/mL)** | 41.0 ± 22.9 | 35.6 ± 20.3 | 0.8 |
| **Serum 25(OH)D (ng/mL)** | 5.9 ± 5.6 | 13.3 ± 8.7 | <0.001 |
| **VDR mRNA copies in PBMC/10^6^ GAPDH mRNA copies** | 87.1 ± 39.5 | 160 ± 57.9 | <0.001 |

**Table 2:** Baseline biochemical parameters of patients with MDR-TB and normal controls; MDR-TB=multi-drug resistant pulmonary tuberculosis, SD= standard deviation, iPTH, intact parathyroid hormone. *Corrected calcium (mg/dL)= measured total calcium (mg/dL) + 0.8 (4.4- serum albumin (g/dL), where 4.4 represent the average albumin level, PBMC= Peripheral blood mono nuclear cells**.**


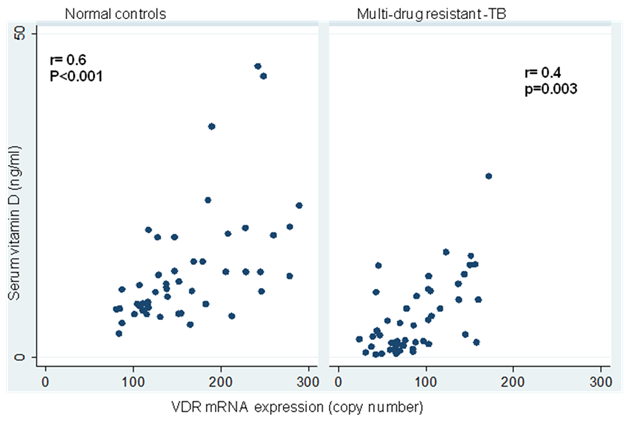


**Figure 2** Scatter plot showing correlation between serum vitamin D and mRNA levels in patients with MDR-TB and normal controls


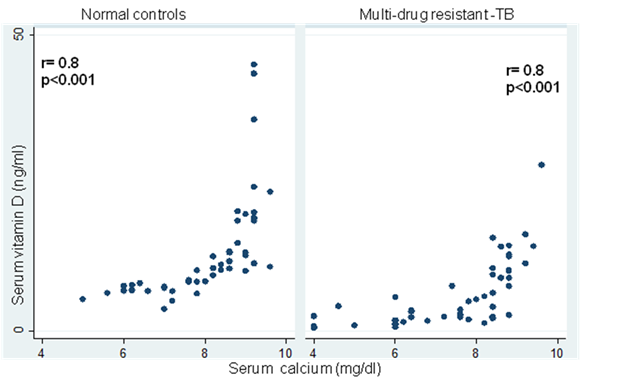


**Figure 3** Scatter plot showing correlation between serum vitamin D and serum calcium levels in patients with MDR-TB and normal controls

VDR mRNA levels were significantly associated with *FokI* VDR genotype in patients with MDR-TB (p=0.02 global), FF and ff genotype (p=0.003), and *Ff* and *ff* genotypes (p=0.01). Tables 3 and 3.1 present both unadjusted and adjusted associations of VDR gene polymorphisms (BsmI, TaqI, FokI) with serum vitamin D levels and VDR mRNA expression in MDR-TB patients and healthy controls. While BsmI and TaqI polymorphisms showed no significant associations, the FokI variant demonstrated a significant relationship in the MDR-TB group. Specifically, individuals with the ff genotype had markedly lower serum 25(OH)D levels and VDR mRNA expression. These associations remained significant even after adjusting for confounders, suggesting that the FokI polymorphism may have a biologically relevant impact on VDR function and immune regulation in tuberculosis pathogenesis. However, no significant association of the VDR genotype (*Bsm*I*, Taq*I and *Fok*I) with mRNA levels was found in the normal control group. Although Table 4 represents the genotypic and allelic frequency distribution of VDR gene (Taq1, Fok1, and Bsm1) polymorphisms in MDRTB and healthy control, no statistically significant association was found between the two groups.

**Multivariate and univariate Analysis Adjusted for Age, Sex, and BMI**

Multivariate analysis using Wilks’ Lambda (Table 5) revealed that among the variables tested, only the Group variable showed a statistically significant multivariate effect on the dependent variables (Wilks’ Lambda = 0.517, F(6, 89) = 13.871, p < 0.001), indicating a strong group-based difference across the outcome measures. The Intercept was also significant (p = 0.045), reflecting overall model fit. In contrast, Age (p = 0.347), Sex (p = 0.193), and BMI (p = 0.096) did not exhibit significant multivariate effects, suggesting that these factors did not meaningfully influence the combination of dependent variables in this analysis.

Univariate regression analyses were also conducted to identify significant predictors for each dependent variable, with results summarized in Table 6. The variable vitamin D was significantly influenced by both Group (p = 0.013) and Sex (p = 0.020), explaining 27.1% of the variance (R² = 0.271). For mRNA, Group emerged as a strong predictor (p = 0.002), accounting for 37.6% of the variance. The model for iPTH showed moderate significance with contributions from Intercept (p = 0.008) and BMI (p = 0.027), but explained a relatively low proportion of variance (R² = 0.093). In contrast, ionized calcium and total calcium were both strongly influenced by Group (p < 0.001), with R² values of 0.435 and 0.436, respectively, indicating a high level of explained variance. Similarly, corrected serum calcium was significantly predicted by both Group (p < 0.001) and BMI (p = 0.009), with the highest explained variance among the outcomes (R² = 0.484). These findings underscore the prominent role of Group as a consistent predictor across multiple outcome measures.

**Table 3** Association of mRNA levels with VDR genotype in patients with MDR-TB and normal controls;

| **Polymorphism** | **Group** | **Genotype** | **Vitamin D**  **(ng/mL ± SD)** | **VDR mRNA Expression**  **(AU ± SD)** | **Global p-value** | **Post-hoc Comparisons**  **(p-value)** |
| --- | --- | --- | --- | --- | --- | --- |
| **BSM I** | MDR-TB (n=50) | BB | 95.3 ± 44.8 | 1.12 ± 0.34 | 0.13 | BB vs Bb: 0.99  BB vs bb: 0.99  Bb vs bb: 0.99 |
|  | Controls (n=50) | BB | 151.4 ± 51.3 | 1.67 ± 0.39 | 0.60 | BB vs Bb: 0.99  BB vs bb: 0.37  Bb vs bb: 0.99 |
|  |  | Bb | 84.5 ± 26.3 /  156.2 ± 57.6 | 1.01 ± 0.29 /  1.61 ± 0.42 |  |  |
|  |  | bb | 73.6 ± 39.5 /  178.4 ± 66.6 | 0.93 ± 0.27 /  1.70 ± 0.46 |  |  |
| **Taq I** | MDR-TB (n=50) | TT | 84.6 ± 37.8 | 1.06 ± 0.31 | 0.81 | TT vs Tt: 0.99  TT vs tt: 0.99  Tt vs tt: 0.99 |
|  | Controls (n=50) | TT | 162.0 ± 56.3 | 1.71 ± 0.36 | 0.90 | TT vs Tt: 0.99  TT vs tt: 0.99  Tt vs tt: 0.99 |
|  |  | Tt | 90.3 ± 43.7 /  155.8 ± 61.4 | 1.02 ± 0.27 /  1.69 ± 0.41 |  |  |
|  |  | tt | 87.7 ± 39.1 /  163.1 ± 62.4 | 1.01 ± 0.24 /  1.75 ± 0.43 |  |  |
| **Fok I** | MDR-TB (n=50) | FF | 100.0 ± 39.0 | 1.25 ± 0.22 | 0.02 | FF vs Ff: 0.88  FF vs ff: 0.003  Ff vs ff: 0.01 |
|  | Controls (n=50) | FF | 156.5 ± 51.2 | 1.78 ± 0.33 | 0.61 | FF vs Ff: 0.99  FF vs ff: 0.59  Ff vs ff: 0.39 |
|  |  | Ff | 88.4 ± 36.3 /  170.6 ± 66.3 | 0.97 ± 0.19 /  1.81 ± 0.35 |  |  |
|  |  | ff | 36.0 ± 8.8 /  80.1 | 0.73 ± 0.17 /  1.52 ± 0.28 |  |  |

Vitamin D levels and VDR mRNA expression are presented as mean ± SD. Expression data units are arbitrary units (AU), normalized to internal control (e.g., GAPDH or β-actin).Global p-values represent overall group differences (e.g., one-way ANOVA).Post-hoc p-values reflect pairwise genotype comparisons with correction (e.g., Bonferroni or Tukey’s). Slash (“/”) indicates values for MDR-TB / Controls, respectively.

Table 3.1: Adjusted Association of VDR Gene Polymorphisms with Serum Vitamin D Levels and VDR mRNA Expression in MDR-TB and Control Groups

| **Polymorphism** | **Group** | **Genotype** | **Adjusted Vitamin D (ng/mL ± SE)** | **Adjusted VDR mRNA Expression (AU ± SE)** | **Global p-value** | **Pairwise Comparisons (Adjusted p-value)** |
| --- | --- | --- | --- | --- | --- | --- |
| **BSM I** | MDR-TB | BB | 93.2 ± 6.8 | 1.10 ± 0.05 | 0.09 | BB vs Bb: 0.97BB vs  bb: 0.91Bb vs bb: 0.99 |
|  | Controls | BB | 148.1 ± 7.2 | 1.65 ± 0.06 | 0.55 | BB vs Bb: 0.95BB vs  bb: 0.35Bb vs bb: 0.97 |
|  |  | Bb | 85.9 ± 5.9 /  154.4 ± 7.1 | 1.00 ± 0.04 /  1.60 ± 0.05 |  |  |
|  |  | bb | 74.5 ± 6.4 /  177.2 ± 7.5 | 0.91 ± 0.04 /  1.69 ± 0.06 |  |  |
| **Taq I** | MDR-TB | TT | 85.1 ± 5.7 | 1.05 ± 0.04 | 0.79 | TT vs Tt: 0.99TT vs  tt: 0.99Tt vs tt: 0.99 |
|  | Controls | TT | 159.2 ± 6.5 | 1.69 ± 0.06 | 0.88 | TT vs Tt: 0.99TT vs  tt: 0.99Tt vs tt: 0.99 |
|  |  | Tt | 89.7 ± 6.1 /  153.6 ± 6.7 | 1.01 ± 0.04 /  1.67 ± 0.05 |  |  |
|  |  | tt | 88.1 ± 5.8 /  161.7 ± 6.8 | 1.00 ± 0.04 /  1.72 ± 0.06 |  |  |
| **Fok I** | MDR-TB | FF | 98.1 ± 6.4 | 1.22 ± 0.04 | 0.01 | FF vs Ff: 0.87FF vs  ff: 0.002Ff vs ff: 0.009 |
|  | Controls | FF | 153.2 ± 6.6 | 1.75 ± 0.05 | 0.58 | FF vs Ff: 0.96FF vs  ff: 0.48Ff vs ff: 0.41 |
|  |  | Ff | 90.5 ± 5.9 /  169.8 ± 6.9 | 0.95 ± 0.03 /  1.78 ± 0.06 |  |  |
|  |  | ff | 38.2 ± 3.2 /  82.1 ± 5.2 | 0.70 ± 0.03 /  1.50 ± 0.05 |  |  |
|  |  |  |  |  |  |  |

Values are **least square means ± standard error (SE)** from ANCOVA models adjusted for **age, sex, and BMI**. **Global p-values** from ANCOVA indicate significance across all genotypes. **Post-hoc p-values** are pairwise comparisons adjusted for covariates and corrected using Bonferroni method. AU: Arbitrary Units (normalized VDR mRNA expression); Vitamin D in ng/mL. Separate ANCOVA models were applied to each polymorphism within MDR-TB and control groups.

**Table 4** Genotypic and allelic frequency distribution of VDR gene (*Taq1, Fok1 and Bsm1*) polymorphism in MDRTB and Healthy control

| VDR Polymorphism Genotype | Healthy Controls (n=50) n% | MDR-TB (n=50) n% | OR (95% CI) | P Value* |
| --- | --- | --- | --- | --- |
| Bsml Genotypic Frequency |  |  |  |  |
| BB | 12 (24) | 17 (34) | 0.61 (0.26-1.47) | 0.27 |
| Bb | 27 (54) | 20 (40) | 1.76 (0.80-3.89) | 0.16 |
| bb | 11 (22) | 13 (26) | 0.8 (0.32-2.02) | 0.63 |
| Bsml Allelic Frequency ‡ |  |  |  |  |
| B | 26 (51) | 27 (54) | 0.92 (0.42-2.02) | 0.84 |
| b | 24 (49) | 23 (46) | 1.08 (0.49-2.38) | 0.84 |
| Taql Genotypic Frequency |  |  |  |  |
| TT | 24 (48) | 21 (42) | 1.27 (0.58-2.81) | 0.54 |
| Tt | 20 (40) | 20 (40) | 1 (0.45-2.23) | 1.0 |
| tt | 6 (12) | 9 (18) | 0.62 (0.2-1.9)) | 0.4 |
| Taql Allelic Frequency ‡ |  |  |  |  |
| T | 34 (68) | 31 (62) | 1.3 (0.57-2.97) | 0.52 |
| t | 16 (32) | 19 (38) | 0.77 (0.34-1.75) | 0.52 |
| Fokl Genotypic Frequency |  |  |  |  |
| FF | 29 (58) | 21 (42) | 1.91 (0.86-4.22) | 0.10 |
| Ff | 19 (38) | 25 (50) | 0.61 (0.25-1.36) | 0.22 |
| ff | 2 (4) | 4 (8) | 0.48 (0.08-2.74) | 0.49 |
| Fokl Allelic Frequency ‡ |  |  |  |  |
| F | 39 (78) | 34 (68) | 0.63 (0.21-1.87) | 0.26 |
| f | 11 (22) | 16 (32) | 0.6 (0.24-1.47) | 0.26 |

* P < 0.05 was considered statistically significant, ‡ Allelic frequency is double the genotypic frequency (2n). VDR = vitamin D receptor; MDR-TB = multidrug-resistant tuberculosis; OR = odds ratio; CI = confidence interval

Table 5: Multivariate Analysis of Variance (MANOVA) Results Using Wilks’ Lambda to Assess the Effects of Age, Sex, BMI, and Group on the Dependent Variables.

Effect Wilks' Lambda F df Sig. (p-value) Interpretation

Intercept 0.868 2.256 6, 89 0.045 Significant

Age 0.929 1.138 6, 89 0.347 Not Significant

Sex 0.909 1.484 6, 89 0.193 Not Significant

BMI 0.889 1.861 6, 89 0.096 Not Significant

Group 0.517 13.871 6, 89 <0.001 Highly Significant

Effect-Th**e independent variable** or **factor** whose influence is being tested on the dependent variable(s); Wilks' Lambda-A **multivariate test statistic** used in MANOVA (Multivariate Analysis of Variance); measures how well the model separates groups; F-The F-**statistic**, used to determine the significance of the overall model (ratio of explained variance to unexplained variance); **df-Degrees of Freedom,** representing the number of independent values that can vary in the analysis; Sig. (p-value)- **Significance level (p-value),** which indicates whether the observed effect is statistically significant (commonly significant if p < 0.05)

Table 6: Summary of Significant Predictors and Explained Variance (R²) for Each Dependent Variable in the Univariate Regression Analysis

| Dependent Variable | Significant Predictors (p < 0.05) | R² Value | Interpretation |
| --- | --- | --- | --- |
| Vit D | Group (p = 0.013),  Sex (p = 0.020) | 0.271 | Group & sex influence vitamin D levels |
| mRNA | Group (p = 0.002) | 0.376 | Group significantly influences mRNA |
| iPTH | Intercept (p = 0.008),  BMI (p = 0.027) | 0.093 | Moderate significance |
| ioz_cal | Group (p < 0.001),  Intercept (p = 0.036) | 0.435 | Strongly influenced by group |
| Total scal | Group (p < 0.001),  Intercept (p = 0.035) | 0.436 | Strong group effect |
| corr_scal | Group (p < 0.001),  BMI(p = 0.009) | 0.484 | Strong group and BMI effect |

Vit D = Serum 25-hydroxyvitamin D levels; mRNA = Expression levels of target messenger RNA; ipth = Intact Parathyroid Hormone; ioz_cal = Ionized Calcium; scal = Total Serum Calcium; corr_scal = Corrected Serum Calcium;R² Value indicates the proportion of variance in the dependent variable explained by the model; p < 0.05 considered statistically significant; values in parentheses represent p-values for each predictor included in the model; Only predictors with p < 0.05 are statistically Significant.

**Discussion**

To our knowledge, this is the first study to explore VDR mRNA expression and its association with vitamin D levels and VDR genotypes in patients with MDR-TB compared to normal controls.

The finding from the current study, MDR-TB patients had considerably lower mean serum 25-hydroxyvitamin D concentrations, and their VDR mRNA expression was 0.6 times lower than that of normal controls. Additionally, we observed that the FokI VDR genotype in MDR-TB patients had a strong association with their VDR mRNA levels. The increased rate of MDR-TB coincided with the development of vitamin D deficiency, probably due to malnutrition and decreased sunlight exposure. A meta-analysis of the association between low vitamin D levels and active tuberculosis reported that low serum vitamin D levels are associated with an increased risk of active tuberculosis (14). Previous studies associated with an increased risk of TB and vitamin D deficiency have only investigated vitamin D levels only (10,15,16). In this study, serum calcium (ionized and total) and intact PTH levels were performed on both subjects to rule out these as confounding factors, and a significantly positive and negative correlation was found with vitamin D levels, respectively, which was as expected in patients without disorders affecting calcium homeostasis.

The VDR gene, located on chromosome 12q, consists of 14 exons. The 5′ untranslated region has six of them (35). The ligand-dependent transcription factor VDR is a member of the nuclear hormone receptor family. When it binds 1,25-(OH)₂D₃, it undergoes a conformational shift, forms a heterodimer with the Retinoid X Receptor (RXR), and enters the nucleus. By attaching to Vitamin D Responsive Elements (VDREs) in gene promoters, this complex regulates transcription. VDR can also indirectly alter gene expression by interfering with transcription factors such NFAT, NF-κB, and AP-1 (36). Functionally, 1,25-(OH)₂D₃ promotes Th2 cytokines (IL-4, TGF-β1) and inhibits Th1 cytokines (IL-2, IFN-γ, GM-CSF) to improve immune modulation and naïve CD4+ cell growth. Additionally, it regulates HLA class II expression (35, 36).

In the present study, we found that the *FokI* polymorphism was significantly associated with VDR mRNA levels. It has been shown that, the VDR is expressed in large number of tissues, it is not surprising that ligand-activated VDR modulates the expression of many genes (35). The *FokI* polymorphism results in the incorporation of three extra amino acids in the NH2 terminus of the VDR protein, which influences transcriptional activity by modulating the interaction with transcription factor IIB (TFIIB) (37,38). Alternatively, the *FokI* polymorphism interferes with the formation of VDR heterodimers with the retinoid X receptor, forming effector complexes (39). However, no such events have been reported for *BsmI* and the *TaqI* polymorphisms, which are located between exons 8 and 9 and exon 9, respectively. It has been reported that the *FokI* genotype contributes to feedback control of the expression of the 25-hydroxyvitamin D-1—hydroxylase (CYP27B1) gene, which is located in the same chromosomal region as the VDR gene and is active in PBMCs (40,41). Vitamin D deficiency leads to an increase in PBMC production by activated vitamin D (42). In accordance with the much lower serum calcium and vitamin D levels, MDR-TB patients exhibited a significantly higher mean serum iPTH content than the other two groups, as reported by Rathored et al. (2023). This implies that the outcomes are not the consequence of an endocrine problem because it appears to represent typical physiological reactions. PTB Cat I patients had a greater sun exposure index (SEI) than the MDR-TB and healthy control groups, which were comparable. Although the higher exposure in PTB Cat I patients compared to healthy controls may cast doubt on this association, this often showed a positive correlation with serum 25(OH)D levels, suggesting, as other studies have shown, that food may be a stronger predictor of the lower serum levels in PTB Cat I (43).

In present research, we assessed serum vitamin D levels and important functional variants of the VDR gene in patients with multidrug-resistant (MDR) TB, filling a crucial knowledge gap on host variables affecting the course of the disease. Considering the ongoing worldwide public health threat of tuberculosis and the increasing incidence of medication resistance, analyzing host immunogenetics offers crucial information about the mechanisms that could affect treatment outcomes and infection susceptibility (44) By influencing the synthesis of antimicrobial peptides and activating macrophages, vitamin D and its receptor are essential components of the immune response against Mycobacterium tuberculosis (45,46). Examining the effects of VDR polymorphisms on receptor expression and function in relation to vitamin D levels, particularly in individuals with MDR-TB, can help advance disease control tactics and open the door to more individualized treatments.

Interestingly, we observed a significant variation in VDR mRNA levels with the *FokI* polymorphism. This study demonstrates that *FokI* genotype may contribute to the expression of VDR mRNA. In addition, homozygosity for the VDR *FokI f* genotype was associated with lower levels of PBMC VDR mRNA expressions in MDR-TB patients than in normal controls. This reflects differences in the effects of ligand-activated receptors between different tissues in patients and controls. We also found that serum vitamin D levels were directly correlated with PBMC VDR mRNA levels and serum calcium levels (total and ionized) in both groups (MDR-TB and normal controls), suggesting that VDR is a ligand-dependent transcription factor and that the ligand for VDR is Vitamin D3, that is, 1,25-(OH)_2_ D_3_ which mediates its biological actions through VDR.

Our results imply that there is no causal relationship between the optimal vitamin D level and the VDR mRNA copy number. Rather, even within the insufficient range, we found statistical correlations between VDR mRNA expression and relative serum 25(OH)D levels. Research shows that VDR expression and immune responses can still be modulated by minor variations in suboptimal vitamin D levels (45,47). Vitamin D, for example, can increase antimicrobial peptides through VDR (45), and slight changes in 25(OH)D affect VDR expression specific to T cells (47). Crucially, we concentrated on FokI polymorphisms, which, independent of vitamin D status, influence VDR mRNA levels and receptor function (33,48). Lower mRNA expression is associated with FokI ff genotypes, which also yield less active receptor isoforms. Despite the low vitamin D levels of the controls, we examined variations by genotype. Collectively, these results which are corroborated by earlier research validate our methodology and imply that VDR transcription is strongly impacted by both genetic and relative vitamin D differences.

Present research lends credibility to the idea that both VDR genotype and vitamin D level affect VDR mRNA expression. In particular, even though there were no appreciable variations in the genotype frequencies between cases and controls, we found that MDR-TB patients with the FokI ff genotype had much decreased VDR mRNA copy numbers (Table 4). This implies that host susceptibility and immune regulation may be more significantly influenced by the functional impact of VDR polymorphisms than by genotype distribution alone. This interpretation is supported by earlier research. For instance, a study conducted in the UK (10) discovered correlations between the severity of tuberculosis and vitamin D responsiveness, but no discernible variations in genotype frequency. VDR SNPs also associated more with cytokine profiles and immunological markers than with the development of disease, according to other research (48).Based to this study results, MDR-TB patients had 0.6 times lower VDR mRNA expression than normal controls. Additionally, there seems to be ethnic variation in the relationship between VDR polymorphisms and TB. South African and Indian populations showed relationships, although some Chinese and European studies found no significant allele differences (21, 48, 49).

This could be the result of impaired VDR signalling, which could lead to elevated inflammation by causing inflammatory cytokines to be expressed more frequently. Our findings in MDR-TB patients are further supported by the fact that vitamin D supplementation improves immunity against tuberculosis by favourably modulating the production of cathelicidin (50). Remarkably, Selvaraj et al. reported lower levels of VDR protein and higher levels of plasma 1,25-(OH)2 D3 (50). Also, the study conducted by Rathored J et al. 2023 found a significant association in MDR-TB and DS-TB patients in context to dietary profile with vitamin D levels and sunlight exposure (51).

In contrast to previous research that suggests the FokI ff genotype produces a longer, less active VDR protein that compromises transcriptional efficiency and immunological function, our data show a robust correlation between the genotype and decreased VDR mRNA expression in MDR-TB patients. We found a favorable connection between 25(OH)D levels and VDR mRNA expression, despite generally low serum vitamin D levels. This suggests that vitamin D can enhance VDR-mediated transcription even at suboptimal levels. The lack of discernible variations in genotype frequencies between cases and controls emphasizes how crucial polymorphisms' functional effects are in assessing TB susceptibility rather than their prevalence. .

There are various limitations on this study. First, since anti-TB therapies are known to reduce vitamin D levels, results may be skewed by the exclusion of drug-susceptible TB patients and the previous anti-TB treatment in MDR-TB cases (14). This suggests that a lack of vitamin D may put patients at risk for re-infection with MDR-TB and worsening of their condition. Second, we did not evaluate other variants such as ApaI, but we concentrated on the FokI polymorphism because of its known functional significance in VDR activity. Third, even after controlling for confounders, the statistical power and generalizability of our findings are severely constrained by the limited number of healthy controls. To confirm and build on our findings, future research should incorporate more VDR polymorphisms and bigger, more representative cohorts. This study presents major new findings: a significant association between *FokI* VDR polymorphisms and low PBMC VDR mRNA levels in MDR-TB patients compared with normal controls. This adds to much recent work on the VDR gene, vitamin D, VDR mRNA levels and MDR-TB, and suggests that the *FokI* (especially mutant allele *f* and homozygous *ff*) and low serum vitamin D levels may be linked to low VDR mRNA levels and increased susceptibility to MDR-TB. This indicates a need for further study in this area, including confirmation of these results using a proteomics approach, and in vitro studies were incorporated into trials of vitamin D supplementation in MDR-TB cases.

**Abbreviation-** VDR- Vitamin D receptor, PBMC- Peripheral blood mono nuclear cells, MDR- Multi drug Resistant, VDRE-Vitamin D Responsive elements, Retinoid X Receptor- RXR, NFAT-Nuclear factor of activated T-cells

**Declarations**

**Ethics approval and consent to participate-** The study was approved by the Institutional Ethical committee of All India Institute of Medical Sciences (IEC-AIIMS) Reference Number: A-08/5.5, New Delhi, India, and written informed consent was obtained from all subjects, including patients and normal controls. Ethics committee (AIIMS-IEC) approved the procedure involved in the present research.

**Clinical Trial**: Not applicable.

**Consent for publication-** All authors agreed and give their consent for publication

**Availability of data and material-** The data that support the findings of this study are available from the corresponding author, [Dr Jaishriram Rathored], upon reasonable request.

**Competing interests-** None (The authors declare no competing interests)

**Funding-** The study was funded by the Department of Biotechnology, Ministry of Science and Technology, Government of India, New Delhi (GRANT NO BT/PR/7898/Med/14/1179)

**Authors' contributions** – **JR:** conceptualization, methodology, data collection, visualization, investigation and writing of original draft and editing; **SKS:** study supervision and review of the original draft and gave his expert advice during the study tenure. **VS:** helps in statistical analysis; **AKS:** helps in biochemical investigation and reviewing the original draft. All authors have reviewed and approved the final draft of the manuscript.

**Acknowledgements-** We are grateful to all study participants and the Department of Medicine staff at AIIMS, New Delhi, for making the study a success.

**REFERENCES**

1. Global Tuberculosis Report 2022 [Internet]. [cited 2025 Mar 12]. Available from: https://www.who.int/teams/global-tuberculosis-programme/tb-reports/global-tuberculosis-report-2022

2. Yin D, Zhong Y, Ling S, Lu S, Wang X, Jiang Z, et al. Dendritic-cell-targeting virus-like particles as potent mRNA vaccine carriers. Nat Biomed Eng. 2025 Feb;9(2):185–200.

3. Wang H, Li B, Sun Y, Ma Q, Feng Y, Jia Y, et al. NIR-II AIE Luminogen-Based Erythrocyte-Like Nanoparticles with Granuloma-Targeting and Self-Oxygenation Characteristics for Combined Phototherapy of Tuberculosis. Adv Mater. 2024 Sep;36(38):e2406143.

4. Sharma S, Rathored J, Ghosh B, Sharma SK. Genetic polymorphisms in TNF genes and tuberculosis in North Indians. BMC Infect Dis [Internet]. 2010 Jun 10 [cited 2025 Mar 12];10:165. Available from: https://www.ncbi.nlm.nih.gov/pmc/articles/PMC2894837/

5. Wang W, Wang J, Hu Z, Yan X, Gao Q, Li X, et al. Advancing Aggregation-Induced Emission-Derived Biomaterials in Viral, Tuberculosis, and Fungal Infectious Diseases. Aggregate [Internet]. [cited 2025 Mar 12];n/a(n/a):e715. Available from: https://onlinelibrary.wiley.com/doi/abs/10.1002/agt2.715

6. Lin X, Yu T, Zhang L, Chen S, Chen X, Liao Y, et al. Silencing Op18/stathmin by RNA Interference Promotes the Sensitivity of Nasopharyngeal Carcinoma Cells to Taxol and High-Grade Differentiation of Xenografted Tumours in Nude Mice. Basic Clin Pharmacol Toxicol. 2016 Dec;119(6):611–20.

7. Zhang X, Zhang Y, Xia W, Liu Y, Mao H, Bao L, et al. The relationship between vitamin D level and second acid-fast bacilli (AFB) smear-positive during treatment for TB patients was inferred by Bayesian network. PLoS One. 2022;17(5):e0267917.

8. Rathored J, Sharma SK, Banavaliker JN, Sreenivas V, Srivastava AK. Response to treatment and low serum vitamin D levels in North Indian patients with treatment-naive category I and multi-drug resistant pulmonary tuberculosis. Ann Med [Internet]. [cited 2025 Mar 12];56(1):2407066. Available from: https://www.ncbi.nlm.nih.gov/pmc/articles/PMC11421155/

9. Selvaraj P, Alagarasu K, Harishankar M, Vidyarani M, Narayanan PR. Regulatory region polymorphisms of vitamin D receptor gene in pulmonary tuberculosis patients and normal healthy subjects of south India. Int J Immunogenet. 2008 Jun;35(3):251–4.

10. Wilkinson RJ, Llewelyn M, Toossi Z, Patel P, Pasvol G, Lalvani A, et al. Influence of vitamin D deficiency and vitamin D receptor polymorphisms on tuberculosis among Gujarati Asians in west London: a case-control study. Lancet. 2000 Feb 19;355(9204):618–21.

11. Bornman L, Campbell SJ, Fielding K, Bah B, Sillah J, Gustafson P, et al. Vitamin D receptor polymorphisms and susceptibility to tuberculosis in West Africa: a case-control and family study. J Infect Dis. 2004 Nov 1;190(9):1631–41.

12. Morrison NA, Qi JC, Tokita A, Kelly PJ, Crofts L, Nguyen TV, et al. Prediction of bone density from vitamin D receptor alleles. Nature. 1994 Jan 20;367(6460):284–7.

13. Rathored J, Sharma SK, Singh B, Banavaliker JN, Sreenivas V, Srivastava AK, et al. Risk and outcome of multidrug-resistant tuberculosis: vitamin D receptor polymorphisms and serum 25(OH)D. Int J Tuberc Lung Dis. 2012 Nov;16(11):1522–8.

14. Nnoaham KE, Clarke A. Low serum vitamin D levels and tuberculosis: a systematic review and meta-analysis. Int J Epidemiol. 2008 Feb;37(1):113–9.

15. Davies PD, Brown RC, Woodhead JS. Serum concentrations of vitamin D metabolites in untreated tuberculosis. Thorax. 1985 Mar;40(3):187–90.

16. Sita-Lumsden A, Lapthorn G, Swaminathan R, Milburn HJ. Reactivation of tuberculosis and vitamin D deficiency: the contribution of diet and exposure to sunlight. Thorax. 2007 Nov;62(11):1003–7.

17. Lewis SJ, Baker I, Davey Smith G. Meta-analysis of vitamin D receptor polymorphisms and pulmonary tuberculosis risk. Int J Tuberc Lung Dis. 2005 Oct;9(10):1174–7.

18. Motsinger-Reif AA, Antas PRZ, Oki NO, Levy S, Holland SM, Sterling TR. Polymorphisms in IL-1beta, vitamin D receptor Fok1, and Toll-like receptor 2 are associated with extrapulmonary tuberculosis. BMC Med Genet. 2010 Mar 2;11:37.

19. Selvaraj P, Chandra G, Kurian SM, Reetha AM, Narayanan PR. Association of vitamin D receptor gene variants of BsmI, ApaI and FokI polymorphisms with susceptibility or resistance to pulmonary tuberculosis. CURRENT SCIENCE. 2003;84(12).

20. Lombard Z, Dalton DL, Venter PA, Williams RC, Bornman L. Association of HLA-DR, -DQ, and vitamin D receptor alleles and haplotypes with tuberculosis in the Venda of South Africa. Hum Immunol. 2006 Aug;67(8):643–54.

21. Babb C, van der Merwe L, Beyers N, Pheiffer C, Walzl G, Duncan K, et al. Vitamin D receptor gene polymorphisms and sputum conversion time in pulmonary tuberculosis patients. Tuberculosis (Edinb). 2007 Jul;87(4):295–302.

22. Roth DE, Soto G, Arenas F, Bautista CT, Ortiz J, Rodriguez R, et al. Association between vitamin D receptor gene polymorphisms and response to treatment of pulmonary tuberculosis. J Infect Dis. 2004 Sep 1;190(5):920–7.

23. Zhou Y, Li Q, Pan R, Wang Q, Zhu X, Yuan C, et al. Regulatory roles of three miRNAs on allergen mRNA expression in Tyrophagus putrescentiae. Allergy. 2022 Feb;77(2):469–82.

24. Zhang YW, Zheng XW, Liu YJ, Fang L, Pan ZF, Bao MH, et al. Effect of Oridonin on Cytochrome P450 Expression and Activities in HepaRG Cell. Pharmacology. 2018;101(5–6):246–54.

25. Pu X, Sheng S, Fu Y, Yang Y, Xu G. Construction of circRNA–miRNA–mRNA ceRNA regulatory network and screening of diagnostic targets for tuberculosis. Ann Med [Internet]. [cited 2025 Mar 12];56(1):2416604. Available from: https://www.ncbi.nlm.nih.gov/pmc/articles/PMC11497567/

26. Treatment of Tuberculosis: Guidelines [Internet]. 4th ed. Geneva: World Health Organization; 2010 [cited 2025 Mar 12]. (WHO Guidelines Approved by the Guidelines Review Committee). Available from: http://www.ncbi.nlm.nih.gov/books/NBK138748/

27. Laboratory services. In: Implementing the WHO Stop TB Strategy: A Handbook for National Tuberculosis Control Programmes [Internet]. World Health Organization; 2008 [cited 2025 Mar 12]. Available from: https://www.ncbi.nlm.nih.gov/books/NBK310753/

28. Khatri GR, Frieden TR. Controlling tuberculosis in India. N Engl J Med. 2002 Oct 31;347(18):1420–5.

29. Zhou Y, Li L, Yu Z, Gu X, Pan R, Li Q, et al. Dermatophagoides pteronyssinus allergen Der p 22: Cloning, expression, IgE-binding in asthmatic children, and immunogenicity. Pediatr Allergy Immunol. 2022 Aug;33(8):e13835.

30. Guo Z, Guan K, Bao M, He B, Lu J. LINC-PINT plays an anti-tumor role in nasopharyngeal carcinoma by binding to XRCC6 and affecting its function. Pathol Res Pract. 2024 Aug;260:155460.

31. Feng C, Wang Y, Xu J, Zheng Y, Zhou W, Wang Y, et al. Precisely Tailoring Molecular Structure of Doxorubicin Prodrugs to Enable Stable Nanoassembly, Rapid Activation, and Potent Antitumor Effect. Pharmaceutics. 2024 Dec 11;16(12):1582.

32. Vupputuri MR, Goswami R, Gupta N, Ray D, Tandon N, Kumar N. Prevalence and functional significance of 25-hydroxyvitamin D deficiency and vitamin D receptor gene polymorphisms in Asian Indians. Am J Clin Nutr. 2006 Jun;83(6):1411–9.

33. Uitterlinden AG, Fang Y, Van Meurs JBJ, Pols HAP, Van Leeuwen JPTM. Genetics and biology of vitamin D receptor polymorphisms. Gene. 2004 Sep 1;338(2):143–56.

34. Arai H, Miyamoto K, Taketani Y, Yamamoto H, Iemori Y, Morita K, et al. A vitamin D receptor gene polymorphism in the translation initiation codon: effect on protein activity and relation to bone mineral density in Japanese women. J Bone Miner Res. 1997 Jun;12(6):915–21.

35. Ogunkolade BW, Boucher BJ, Prahl JM, Bustin SA, Burrin JM, Noonan K, et al. Vitamin D receptor (VDR) mRNA and VDR protein levels in relation to vitamin D status, insulin secretory capacity, and VDR genotype in Bangladeshi Asians. Diabetes. 2002 Jul;51(7):2294–300.

36. van Etten E, Verlinden L, Giulietti A, Ramos-Lopez E, Branisteanu DD, Ferreira GB, et al. The vitamin D receptor gene FokI polymorphism: functional impact on the immune system. Eur J Immunol. 2007 Feb;37(2):395–405.

37. Arai H, Miyamoto K, Taketani Y, Yamamoto H, Iemori Y, Morita K, et al. A vitamin D receptor gene polymorphism in the translation initiation codon: effect on protein activity and relation to bone mineral density in Japanese women. J Bone Miner Res. 1997 Jun;12(6):915–21.

38.Jurutka PW, Remus LS, Whitfield GK, Thompson PD, Hsieh JC, Zitzer H, et al. The polymorphic N terminus in human vitamin D receptor isoforms influences transcriptional activity by modulating interaction with transcription factor IIB. Mol Endocrinol. 2000 Mar;14(3):401–20.

39. Haussler MR, Whitfield GK, Haussler CA, Hsieh JC, Thompson PD, Selznick SH, et al. The nuclear vitamin D receptor: biological and molecular regulatory properties revealed. J Bone Miner Res. 1998 Mar;13(3):325–49.

40. Smith SJ, Rucka AK, Berry JL, Davies M, Mylchreest S, Paterson CR, et al. Novel mutations in the 1alpha-hydroxylase (P450c1) gene in three families with pseudovitamin D-deficiency rickets resulting in loss of functional enzyme activity in blood-derived macrophages. J Bone Miner Res. 1999 May;14(5):730–9.

41. Bell NH. Renal and nonrenal 25-hydroxyvitamin D-1alpha-hydroxylases and their clinical significance. J Bone Miner Res. 1998 Mar;13(3):350–3.

42. Dusso AS, Finch J, Brown A, Ritter C, Delmez J, Schreiner G, et al. Extrarenal production of calcitriol in normal and uremic humans. J Clin Endocrinol Metab. 1991 Jan;72(1):157–64.

43. Goswami R, Mondal AM, Tomar N, Ray D, Chattopadhyay P, Gupta N, et al. Presence of 25(OH)D deficiency and its effect on vitamin D receptor mRNA expression. Eur J Clin Nutr. 2009 Mar;63(3):446–9.

44. Lawn SD, Zumla AI. Tuberculosis. The Lancet [Internet]. 2011 Jul [cited 2025 May 20];378(9785):57–72. Available from: https://linkinghub.elsevier.com/retrieve/pii/S0140673610621733

45. Liu PT, Stenger S, Li H, Wenzel L, Tan BH, Krutzik SR, et al. Toll-like receptor triggering of a vitamin D-mediated human antimicrobial response. Science. 2006 Mar 24;311(5768):1770–3.

46. Martineau AR, Jolliffe DA, Hooper RL, Greenberg L, Aloia JF, Bergman P, et al. Vitamin D supplementation to prevent acute respiratory tract infections: systematic review and meta-analysis of individual participant data. BMJ. 2017 Feb 15;356:i6583.

47. Mora JR, Iwata M, von Andrian UH. Vitamin effects on the immune system: vitamins A and D take centre stage. Nat Rev Immunol. 2008 Sep;8(9):685–98.

48. Selvaraj P, Chandra G, Jawahar MS, Rani MV, Rajeshwari DN, Narayanan PR. Regulatory role of vitamin D receptor gene variants of Bsm I, Apa I, Taq I, and Fok I polymorphisms on macrophage phagocytosis and lymphoproliferative response to mycobacterium tuberculosis antigen in pulmonary tuberculosis. J Clin Immunol. 2004 Sep;24(5):523–32.

49. Zhou Y, Hof S van den, Wang S, Pang Y, Zhao B, Xia H, et al. Association between genotype and drug resistance profiles of Mycobacterium tuberculosis strains circulating in China in a national drug resistance survey. PLOS ONE [Internet]. 2017 Mar 23 [cited 2025 May 20];12(3):e0174197. Available from: https://journals.plos.org/plosone/article?id=10.1371/journal.pone.0174197

50. Selvaraj P, Prabhu Anand S, Harishankar M, Alagarasu K. Plasma 1,25 dihydroxy vitamin D3 level and expression of vitamin d receptor and cathelicidin in pulmonary tuberculosis. J Clin Immunol. 2009 Jul;29(4):470–8.

51. Rathored J, Sharma SK, Chauhan A, Singh B, Banavaliker JN, Sreenivas V, et al. Low serum vitamin D in North Indian multi-drug resistant pulmonary tuberculosis patients: the role of diet and sunlight. Ann Med. 2023;55(2):2291554.
